# Supplementary material for: Bioinspired crowding directs supramolecular polymerisation
Source: Nat Commun. 2023 Feb 25;14:1084. doi: 10.1038/s41467-023-36540-x (PMC9968348; doi:10.1038/s41467-023-36540-x)
Supplement: Supplementary file 1 — Supplementary Information [file 41467_2023_36540_MOESM1_ESM.pdf]

Supplementary Information for:

Bioinspired Crowding directs Supramolecular Polymerization

Nils Bäumer[a], Eduardo Castellanos[b], Bartolome Soberats[b] & Gustavo Fernández[a,]*

[a] Westfälische-Wilhelms Universität Münster, Organisch Chemisches Institut, Corrensstraße 36, 48149 Münster, Germany

[b] Department of Chemistry, University of the Balearic Islands, Cra. Valldemossa, Km. 7.5, Palma de Mallorca 07122, Spain

* e-mail: fernandg@uni-muenster.de

Table of Contents

Supplementary Notes 2

Supplementary Tables 11

- **Unchanged viscosity of MCH with respect to the addition of Agg2 at dilute concentrations** 11
- **Evaluation of the thermodynamic stability of Agg1U and Agg1C** 12

Supplementary Figures 13

- **Analysis of self-sorted self-assembly of 1 and 2 under thermodynamic conditions** 13
- **Self-assembly of 1 and 2 under kinetic conditions in crowded and uncrowded environments** 16
- **Analysis of self-sorted self-assembly of 1 and 2 under kinetic conditions** 24
- **Insights into the mechanism of supramolecular crowding in binary mixtures of 1 and Agg2** 27
- **Evaluation of the thermodynamic stability of Agg1U and Agg1C** 29
- **Time-dependent morphological transformation of Agg1C** 34
- **Seed-induced supramolecular polymerization experiments in crowded and uncrowded environments** 40
- **Self-assembly of 1 in the presence of distinct crowding agents** 47

Supplementary Notes

Supplementary Note 1. Analysis of self-sorted self-assembly of **1** and **2** under thermodynamic conditions

Temperature-dependent cooling experiments of mixtures of **1** and **2** under constant mechanical agitation reveal identical spectroscopic changes as those observed for both compounds in isolation. In short, all absorption bands of **2** exhibit a hypsochromic shift, which is characteristic for the self-assembly process of **2** (Supplementary Figure 1). These changes are independent of the added amount of **1**, while the typical spectroscopic changes associated with the self-assembly of **1** can also be appreciated above a certain concentration, which can overcome the nucleation penalty of the assembly process of **1** (c < 10 x 10^{-6} M). Ultimately, the self-assembly of **2** yields flexible, elongated one-dimensional fibres, matching the morphology previously observed for Agg**2** (Supplementary Figure 2). Furthermore, for those samples in which **1** is present at higher concentrations, the bundled fibres previously observed for Agg**1** (Agg**1U**) can also be observed during AFM analysis, while Agg**2** can be appreciated in the background (Supplementary Figure 2). In addition, the aggregation curves of the self-assembly processes of **1** and **2** obtained from mixed samples match those observed for both compounds in isolation providing further evidence for the narcissistic self-sorting of **1** and **2** (Supplementary Figure 3).

Supplementary Note 2. Self-assembly of **1** and **2** under kinetic conditions in crowded and uncrowded environments

Supplementary Figure 5-12 depict the kinetic self-assembly behaviour of **1** and **2** using various crowded environments. These studies establish some general trends, but also some limitations in employing crowding agents for controlled self-assembly. Some key trends will be shortly discussed in the following. Firstly, it becomes apparent that the concentration of **1** has a much higher influence on the self-assembly kinetics than the amount of crowding agent present in solution. This is particularly obvious when comparing Supplementary Figure 8 and 10, as the lag time is drastically affected when using 0.25 eq. of **2** in solutions containing **1** at a concentration of 20×10^{-6} M (Supplementary Figure 8), while the same relative amount has virtually no influence on the self-assembly kinetics in higher concentrated solutions (Supplementary Figure 10). Secondly, the amount of crowding agent influences the self-assembly of **1** depending on the amount of crowding agent added but gets ultimately overshadowed by increasing concentrations of **1** (Supplementary Figure 10). Additionally, the kinetic influence of the added crowder will not further increase beyond a threshold concentration, from which point onwards increasing amounts of crowding agents have virtually no additional effect (Supplementary Figure 11). Further, even extremely dilute samples of the crowder are able to induce the formation of Agg**1C** with a characteristic increase in lag time (Supplementary Figure 12). This observation strongly suggests that the mechanism by which the crowding agent influences the self-assembly of **1** is attributable to (weak) repulsive interactions, since attractive interactions, such as secondary nucleation events or surface mediated self-assembly, would lead to an acceleration of the self-assembly process upon increasing the amount of crowding agent.

The resulting supramolecular polymer of **1** (Agg**1C**) has a remarkably decreased anisotropic nature compared to Agg**1U**, as corroborated by SEM analysis (Fig. 2) in the dried state, irrespective of the sample preparation (drop casting or spin coating, Supplementary Figure 13+14), as well as by combined DLS and SLS analysis in solution (Supplementary Figure 15). Based on the spectroscopic characterization of the self-assembly of Agg**1C** described above, as well as on the fact that Agg**1C** is far less anisotropic than Agg**1U**, we infer that the mechanism of the crowding is derived from merely steric repulsion between the aggregates in solution. Most likely, the formation of less anisotropic structures reduces the surface-to-volume ratio and can effectively decrease steric interactions between different solutes.

Supplementary Note 3. Analysis of self-sorted self-assembly of **1** and **2** under kinetic conditions

In order to confirm the narcissistic self-assembly of **1** and **2** observed during thermodynamic self-assembly conditions, additional spectroscopic measurements were conducted (Supplementary Figure 16-21). Photoluminescence (Supplementary Figure 16) as well as FT-IR (Supplementary Figure 17) spectroscopic studies of Agg**1C** reveal a nearly perfect summation of the spectra obtained for Agg**2** and Agg**1U** (i.e. in isolation), strongly suggesting a narcissistic self-assembly mechanism of both compounds. Further, XRD analysis of Agg**1U** and Agg**1C** shows a perfect match between the observed diffraction patterns (Fig. 3 and Supplementary Figure 18+19). Considering that Agg**2** has been previously observed to be XRD silent, this strongly suggests that the molecular packing of **1** in Agg**1C** remains identical to that observed for Agg**1U**. A time-dependent AFM analysis of the self-assembly process of Agg**1C** further supports this hypothesis (Supplementary Figure 20). Namely, the self-assembly of Agg**2** can be observed initially, followed by the onset of self-assembly of Agg**1C** (Supplementary Figure 20a,b). Further equilibration leads to the formation of larger hierarchical structures (Supplementary Figure 20c), which can eventually precipitate from the solution to yield Agg**2** as the only supramolecular entity left in solution (Supplementary Figure 20d). NMR analysis of the precipitate as well as the supernatant supports this analysis (Supplementary Figure 21). In the supernatant, a much higher content of **2** compared to **1** could be observed, which indicates that Agg**2** remains present in solution and does not co-assemble/precipitate with **1**. Similarly, an analysis of the precipitate reveals a nearly quantitative removal of **2**, supporting the narcissistic self-assembly, while the small residue of **2** can be readily explained by the imperfect separation of the supernatant from the precipitate using decantation.

Supplementary Note 4. Insights into the mechanism of supramolecular crowding in binary mixtures of **1** and Agg**2**

To probe the interactions between **1** and Agg**2** within the lag time, we conducted additional spectroscopic analysis. Initially, we aimed to investigate absorption of **1** within the lag time to gain more insights into the potential interactions with Agg**2**. To this end, we prepared dilute samples of individual **1** and **2** as well as their mixture (c_1 = 20 x 10^{-6} M; c_2 = 5 x 10^{-6} M) and confirmed the trapping of **1** in the molecularly dissolved state after conducting the kinetic protocol described in the experimental details. The UV/Vis spectra clearly indicate the absence of large assemblies, as can be inferred from the missing optical density in the low energy regime of the spectrum (Supplementary Figure 22). When comparing the absorption properties of **1** in uncrowded and crowded conditions, by subtracting the absorption of Agg**2**, it is clear that the only deviations in the spectra are present in areas where Agg**2** shows a relatively strong absorption (around 370 nm and above 400 nm), possibly owing to small errors in the sample preparation. In contrast, the position of the absorption maximum of **1** remains completely unchanged, indicating no strong chromophore-chromophore interactions in the ground state. As the excited state is generally more sensitive to changes in the chromophore environment, we subjected the same solutions to photoluminescence analysis (Supplementary Figure 22). On the basis of these studies, it is apparent that the emission of **1** in crowded and uncrowded media remains unchanged, which further supports our assignment of the regime to the molecularly dissolved state of **1** with an energetically high nucleation barrier. It is known in the literature (*Angew. Chem. Int. Ed.* **2020**, *59*, 19841; *J. Am. Chem. Soc.* **2021**, *143*, 11777) that interactions between molecularly dissolved species and the surface of a supramolecular polymer can occur, even if the thermodynamically most stable state is a narcissistically self-sorted system. These interactions have been linked to secondary nucleation processes, which accelerate an aggregate formation in the presence of other polymers. Although our observations are in direct contradiction with this behavior, we nevertheless sought to investigate whether **1** could nucleate on the surface of Agg**2**. To this end, we employed DLS studies; however, the experimental results were inconclusive owing to the thin morphology of Agg**2** and insufficient concentration (Supplementary Figure 23). We also wish to reemphasize the time-dependent AFM studies presented in Supplementary Figure 20. It can be clearly observed that during the lag time (a) only Agg**2** can be visualized on the HOPG surface. Afterwards (within the nucleation regime), the formation of two-dimensional plates can be observed, which exhibit significant clustering upon elongation (b,c), suggesting the narcissistic self-assembly of **1**. Following the precipitation of the flower-like assemblies, the isolated Agg**2** can be observed again. Crucially, these structures observed after precipitation of Agg**1C** with regards to the morphology are identical to those observed within the lag time of **1**, highlighting that no accumulation of **1** on the aggregate surface occurs. The same holds true for the fibers of Agg**2** observed within the lag time of **1** under various conditions (see for example Supplementary Figure 6-8). We further investigated if an effect of the supramolecular crowder on the assembly of **1** could be achieved under fast cooling conditions (2 K/min cooling rate, 2 K data interval), i.e. intermediate experimental conditions between the kinetic self-assembly protocol described in the methods section and the cooling under thermodynamic

equilibrium (Supplementary Figure 1). To our satisfaction, a minor increase in the heating/cooling hysteresis is observed in crowded media, although ultimately the influence of the crowder is overshadowed by the continuous cooling enforcing a fast elongation of Agg**1U** (Supplementary Figure 24). These results further support our original assumption that **1** exists as a molecularly dissolved species within the lag time, irrespective of whether this condition is achieved by dilution (uncrowded) or by the addition of a supramolecular crowder. This suggests that the supramolecular crowder can disfavor the formation of the nucleus, in a similar way as a simple dilution. In contrast to the diluted state, the crowded state can also suppress a one-dimensional growth, which we ascribe to repulsive interactions as a consequence of motion within the solvent. As a result, a two-dimensional growth behavior is facilitated, which ultimately leads to the formation of the flower-like particles. Based on our experimental evidence as well as the results disclosed in our earlier analysis of the self-assembly of **1** (ref 38), we attribute the lag time and the hindered nucleation of **1** to the high flexibility of the solubilizing chains in combination with the high steric demand of the central $PtCl_2$ core. This hindered nucleation becomes more pronounced upon dilution and upon the addition of a supramolecular crowder.

Supplementary Note 5. Time-dependent morphological transformation of **Agg1C**

As briefly discussed in the main part, aggregate Agg**1C** can undergo a morphological transformation, which is a relatively rare occurrence in self-assembled structures exhibiting such high hierarchical order. In our time resolved SEM studies depicted in Supplementary Figure 32-35, many self-assembled states showing a morphology between the initial hierarchical flower-like structures and the more ordered nanoribbons can be observed. In these intermediate stages, various degrees of folding can also be appreciated. Two surprising trends could be noticed during this investigation. Firstly, it appears that the morphological transition is limited to low concentrated solutions of **1**, as no nanoribbons could ever be observed for solutions containing 80 x 10^{-6} M of **1**. In fact, the observed hierarchical flower-like structures maintained their shape throughout the entire study, without any signs of folding or rearrangement. This observation can be rationally explained by a kinetic trap exhibited in large hierarchical flower-like structures, as the multitude of intraparticle interactions sufficiently stabilizes the morphology. Secondly, the structural transformation appears to be accelerated by lower amounts of crowding agent, as samples containing very low ratios of crowding agent (<1:40) show no traces of hierarchical flower-like structures after 2 weeks and instead only assemblies in differing stages of folding can be observed. We infer from these observations that both the extended fibre structures of Agg**1U**, as well as larger flower-like structures of Agg**1C**, can represent a kinetic trap in the hierarchical self-assembly process, which efficiently hamper a rearrangement or folding into the more ordered ribbon structure (Agg**1T**).

Interestingly, Agg**1U** does not show any time resolved structural evolution, even in the presence of Agg**2** (Supplementary Figure 36), which supports the conclusion that Agg**1U** represents a hierarchically trapped morphology.

Supplementary Note 6. Seed-induced supramolecular polymerization experiments in crowded and uncrowded environments

Standard seeded supramolecular polymerization protocols (see methods for experimental details) allow an assessment of how this process is translated to crowded self-assembly environments. Initially, the stability of both aggregates towards mechanical agitation using sonication was assessed. Agg**1C**, both in the pristine hierarchical flower-like state, as well as in the incubated nanoribbon state (Agg**1T**), is transformed into smaller hierarchical flower-like structures upon sonication. However, due to the high stability of the two-dimensional nanosheets, a further fragmentation was not observed, even after prolonged agitation (Supplementary Figure 38). Unfortunately, following the seeded protocol did not disclose any morphological change, irrespective of the added aliquots of **1**, as the seeds bundle over time to regain the large hierarchical flower-like structures of Agg**1C** observed prior to sonication. This fact prevents further analysis using Agg**1C** as seeds.

To our delight, this dramatically changes when Agg**1U** is used for seed formation. Sonication of Agg**1U** yields small nanoribbons, similar in morphology to Agg**1T**. These seeds also maintain their structure over time and do not show any bias towards clustered agglomerates. Adding these seeds to aliquots of **1** in crowded conditions leads to an efficient seed-induced polymerization (Agg**1Ts**) and the resulting nanoarchitectures size depends on the amount of added **1**, as expected (Supplementary Figure 41). However, due to the conflicting trends of increased optical density upon elongation and poor colloidal stability of the final product, a two-step time-dependent precipitation precludes detailed kinetic analysis.

Captivatingly, a drastically different seed-induced self-assembly behaviour is observed in uncrowded environments (Supplementary Figure42). Upon addition of aliquots of **1**, an initial decrease in optical density is observed, with a rate depending on the amount of added **1**, due to the formation of larger, poorly solvated architectures. However, this process is overruled over time, as the remaining seeds in solution can diffuse freely, leading to a random distribution of aggregates and in turn unsteady levels of absorbance, indicating that the seeded self-assembly does not affect all seeds identically. SEM analysis confirms these assumptions made based on spectroscopy. In fact, highly elongated morphologies (Agg**1Us**) could be observed together with practically unchanged seeds after the incubation period. This observation suggests that the added seeds can serve as a nucleation side in uncrowded conditions, which then leads to a highly efficient self-acceleration based on secondary effects.

Interestingly, Agg**1Ts** shows a clear increase in hierarchical stability compared to Agg**1C**, as can be seen in VT-UV/Vis experiments at higher concentrations, while lower concentrations, i.e. smaller morphologies, show a comparatively lower hierarchical stability (Supplementary Figure 43). This observation allows us to conclude that the time-dependent transformation of Agg**1C** previously observed in time-resolved SEM (Supplementary Figure 32-35) represents the transformation into a hierarchically more stable structure.

In order to demonstrate that the structure of the molecular stacks is unaffected by this sample preparation protocol, we turned again to XRD analysis of the different samples (Supplementary Figure 44+45). Indeed, the XRD patterns for the aggregates obtained by the seeded approach match perfectly with the aggregates obtained in crowded as well as uncrowded conditions. These results highlight the versatile approach of the sample preparation method towards inducing different supramolecular structures based on the hierarchical self-assembly of the same molecular packing.

Supplementary Note 7. Self-assembly of **1** in the presence of distinct crowding agents

To assess the capabilities of other supramolecular self-assembled structures to act as suitable crowding agents, we followed the same kinetic self-assembly protocol described in the method section. As a general observation, it can be stated that all supramolecular building blocks capable of forming extended one-dimensional architectures are suitable as crowding agents (see Supplementary Figure 47,48,52). In contrast, self-assembled structures forming two-dimensional sheets or nanoparticle structures (see Supplementary Figure 49+50) do not efficiently affect the self-assembly of **1**. Similarly, a covalent polymer (polystyrene) failed to produce Agg**1C** using the same protocol (Supplementary Figure 53). However, one significant deviation from this general trend was observed in our assessment. Compound **7** forms isolated one-dimensional fibres at low concentration, which have a strong tendency to interconnect at elevated concentration leading to the formation of supramolecular gels. Interestingly, the self-assembly kinetics of **1** are nearly unaffected by the presence of **7**, yet the self-assembled morphology under dilute crowding conditions exhibits hierarchical structures reminiscent of Agg**1C**. At higher concentrations of **7**, the self-assembly of **1** yields more isolated two-dimensional sheets and isolated fibre bundles distributed inside the gel matrix of **7** (Supplementary Figure 51). These observations indicate a more complex interaction between Agg**7** and **1**, which hints at interesting prospects of using gelators in crowded self-assembly and will be further elaborated in future studies.

Supplementary Tables

Unchanged viscosity of MCH with respect to the addition of Agg2 at dilute concentrations

Supplementary Table 1: Summary of the rheology data obtained for a sample of pure MCH as reference (shear stress (σ), viscosity (η) and torque (τ)).

Run	T / K	σ / Pa	η / mPa·s	τ / mN·m
1	294.06	0.03300	0.65994	1.3503E-4
2	294.13	0.03146	0.62915	1.2873E-4
3	294.15	0.03280	0.65607	1.3424E-4

Supplementary Table 2: Summary of the rheology data obtained for a sample of Agg**2** in MCH using a concentration of 10 x 10^{-6} M (shear stress (σ), viscosity (η) and torque (τ)).

Run	T / K	σ / Pa	η / mPa·s	τ / mN·m
1	294.13	0.03633	0.68305	1.3976E-4
2	294.15	0.03415	0.69444	1.4209E-4
3	294.15	0.03472	0.65143	1.3329E-4

Supplementary Table 3: Summary of the rheology data obtained for a sample of Agg**2** in MCH using a concentration of 20 x 10^{-6} M (shear stress (σ), viscosity (η) and torque (τ)).

Run	T / K	σ / Pa	η / mPa·s	τ / mN·m
1	294.15	0.03269	0.65372	1.3376E-4
2	294.15	0.03268	0.65350	1.3371E-4
3	294.15	0.03277	0.65539	1.3410E-4

Supplementary Table 4: Summary of the rheology data obtained for a sample of Agg**2** in MCH using a concentration of 100 x 10^{-6} M (shear stress (σ), viscosity (η) and torque (τ)).

Run	T / K	σ / Pa	η / mPa·s	τ / mN·m
1	294.13	0.03960	0.79201	1.6205E-4
2	294.15	0.03983	0.79650	1.6297E-4
3	294.15	0.03948	0.78957	1.6155E-4

Evaluation of the thermodynamic stability of Agg1U and Agg1C

Supplementary Table 5: Thermodynamic parameters obtained from individually fitting the heating curves of Agg**1U** using the nucleation-elongation model.

Concentration / 10^{-6} M	ΔH_e (SD) / kJ mol^{-1}	ΔH_n (SD) / kJ mol^{-1}	ΔS (SD) / kJ mol^{-1}	T_e (SD) / K	K_e / 10^4 M^{-1}	K_n / M^{-1}	σ / 10^{-3}
10	-186.59 (7.52)	-13.03 (1.05)	-0.475 (0.023)	326.98 (0.21)	10.00	827.40	8.27
20	-241.20 (11.71)	-13.54 (1.13)	-0.639 (0.036)	330.69 (0.17)	5.00	362.99	7.26
30	-265.01 (6.62)	-12.31 (0.58)	-0.711 (0.020)	332.41 (0.10)	3.33	387.61	11.63
40	-251.15 (9.76)	-12.94 (0.80)	-0.667 (0.029)	334.35 (0.13)	2.50	238.06	9.52

Supplementary Table 6: Thermodynamic parameters obtained from individually fitting the heating curves of Agg**1C** using the nucleation-elongation model.

Concentration / 10^{-6} M	ΔH_e (SD) / kJ mol^{-1}	ΔH_n (SD) / kJ mol^{-1}	ΔS (SD) / kJ mol^{-1}	T_e (SD) / K	K_e / 10^4 M^{-1}	K_n / 10^2 M^{-1}	σ / 10^{-3}
10 (**1**) 2.5 (**2**)	-492.18 (1.08)	-4.33 (0.01)	-1.414 (0.003)	326.03 (0.03)	10.00	202.510	202.5
20 (**1**) 5 (**2**)	-408.10 (5.03)	-13.51 (0.39)	-1.144 (0.015)	330.79 (0.04)	5.00	3.67	7.23
30 (**1**) 7.5 (**2**)	-488.00 (5.94)	-9.79 (0.25)	-1.381 (0.018)	332.55 (0.03)	3.33	9.64	28.94
40 (**1**) 10 (**2**)	-382.86 (6.12)	-12.89 (0.47)	-1.060 (0.018)	334.51 (0.05)	2.50	2.42	9.70

Supplementary Table 7**:** Comparison between the thermodynamic parameters obtained from individually fitting the heating curves of both aggregates using the nucleation-elongation model and averaging the resulting parameters and the results from global fitting of the denaturation curves of both aggregates using the denaturation model.

Method (Aggregate)	$\Delta G^{298\,K}$ / kJ·mol^{-1}	σ / 10^{-3}
Heating (Agg**1U**)	-50.2	9.2
Heating (Agg**1C**)	-70.2	15.3
Denaturation (Agg**1U**)	-41.5	21.4
Denaturation (Agg**1C**)	-41.2	19.6

Supplementary Figures

Analysis of self-sorted self-assembly of 1 and 2 under thermodynamic conditions

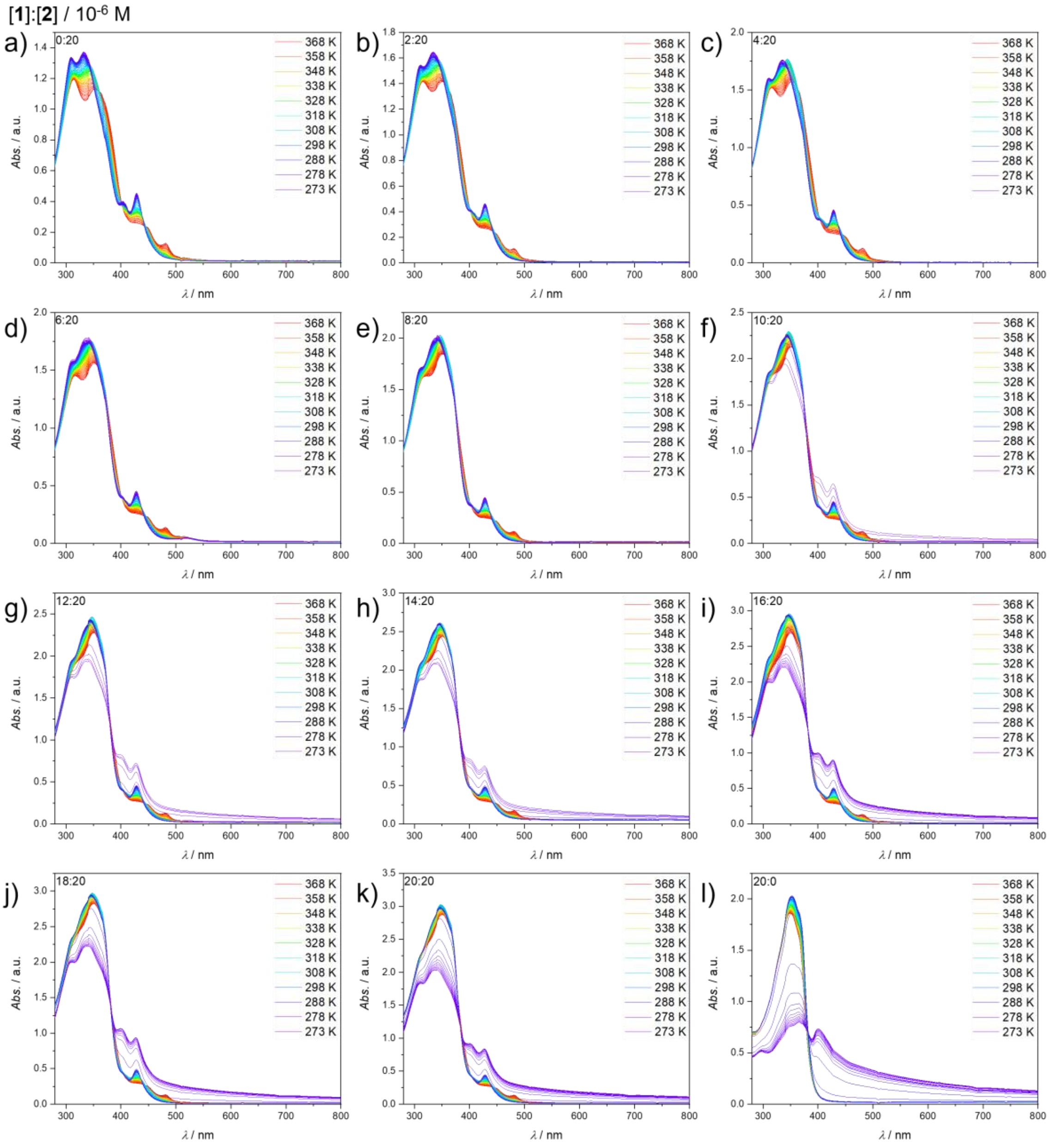

Supplementary Figure 1: a-l) Temperature-dependent UV/Vis absorption spectra of mixtures of **1** and **2** at different ratios (c_1 = 0 x 10^{-6} M; c_2 = 20 x 10^{-6} M (a); c_1 = 2 x 10^{-6} M; c_2 = 20 x 10^{-6} M (b); c_1 = 4 x 10^{-6} M; c_2 = 20 x 10^{-6} M (c); c_1 = 6 x 10^{-6} M; c_2 = 20 x 10^{-6} M (d); c_1 = 8 x 10^{-6} M; c_2 = 20 x 10^{-6} M (e); c_1 = 10 x 10^{-6} M; c_2 = 20 x 10^{-6} M (f); c_1 = 12 x 10^{-6} M; c_2 = 20 x 10^{-6} M (g); c_1 = 14 x 10^{-6} M; c_2 = 20 x 10^{-6} M (h); c_1 = 16 x 10^{-6} M; c_2 = 20 x 10^{-6} M (i); c_1 = 18 x 10^{-6} M; c_2 = 20 x 10^{-6} M (j); c_1 = 20 x 10^{-6} M; c_2 = 20 x 10^{-6} M (k); c_1 = 20 x 10^{-6} M; c_2 = 0 x 10^{-6} M (l)) between 368 K and 273 K, using a cooling rate of 1 K/min, a data interval of 1 K and constant stirring using a magnetic stirring bar at 1600 rpm.

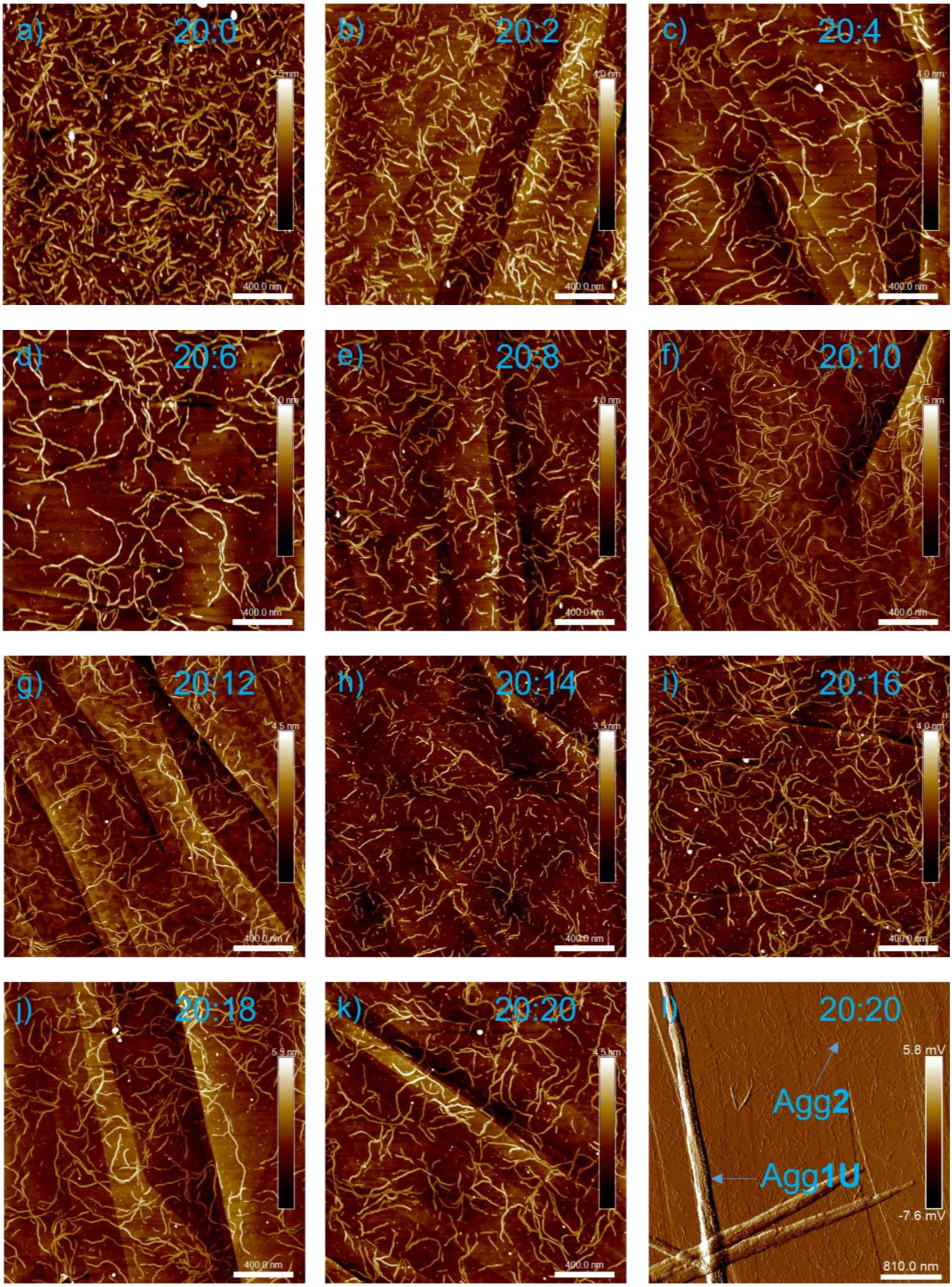

Supplementary Figure 2: a-l) AFM height images recorded for different mixtures of **1** and **2** following the temperature-dependent UV/Vis studies displayed in Supplementary Figure 1 (c_1 = 0 x 10^{-6} M; c_2 = 20 x 10^{-6} M (a); c_1 = 2 x 10^{-6} M; c_2 = 20 x 10^{-6} M (b); c_1 = 4 x 10^{-6} M; c_2 = 20 x 10^{-6} M (c); c_1 = 6 x 10^{-6} M; c_2 = 20 x 10^{-6} M (d); c_1 = 8 x 10^{-6} M; c_2 = 20 x 10^{-6} M (e); c_1 = 10 x 10^{-6} M; c_2 = 20 x 10^{-6} M (f); c_1 = 12 x 10^{-6} M; c_2 = 20 x 10^{-6} M (g); c_1 = 14 x 10^{-6} M; c_2 = 20 x 10^{-6} M (h); c_1 = 16 x 10^{-6} M; c_2 = 20 x 10^{-6} M (i); c_1 = 18 x 10^{-6} M; c_2 = 20 x 10^{-6} M (j); c_1 = 20 x 10^{-6} M; c_2 = 20 x 10^{-6} M (k); c_1 = 20 x 10^{-6} M; c_2 = 20 x 10^{-6} M (l)). The images display narcissistically self-sorted Agg**2**, while only one AFM phase image depicting both aggregates is shown for reference.

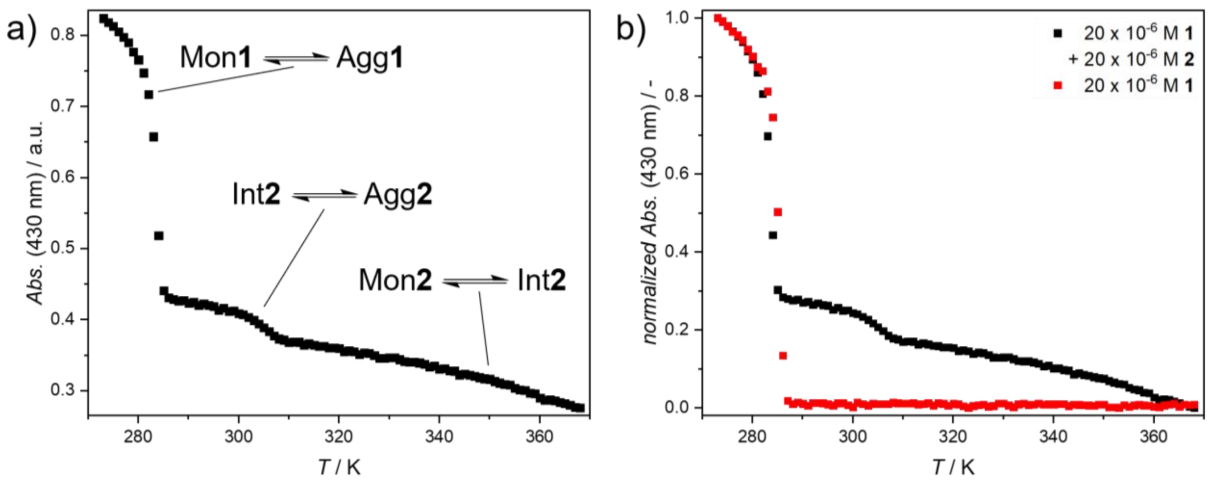

Supplementary Figure 3: a) Representative temperature-dependent cooling curve obtained from the UV/Vis studies in Supplementary Figure 1k (c_1 = 20 x 10^{-6} M; c_2 = 20 x 10^{-6} M), revealing distinct aggregation regimes of **1** and **2**. b) Visual comparison between the temperature-dependent cooling curves of **1** in a mixture with **2** (black) and in isolation (red), revealing an excellent match between both assembly processes.

Self-assembly of 1 and 2 under kinetic conditions in crowded and uncrowded environments

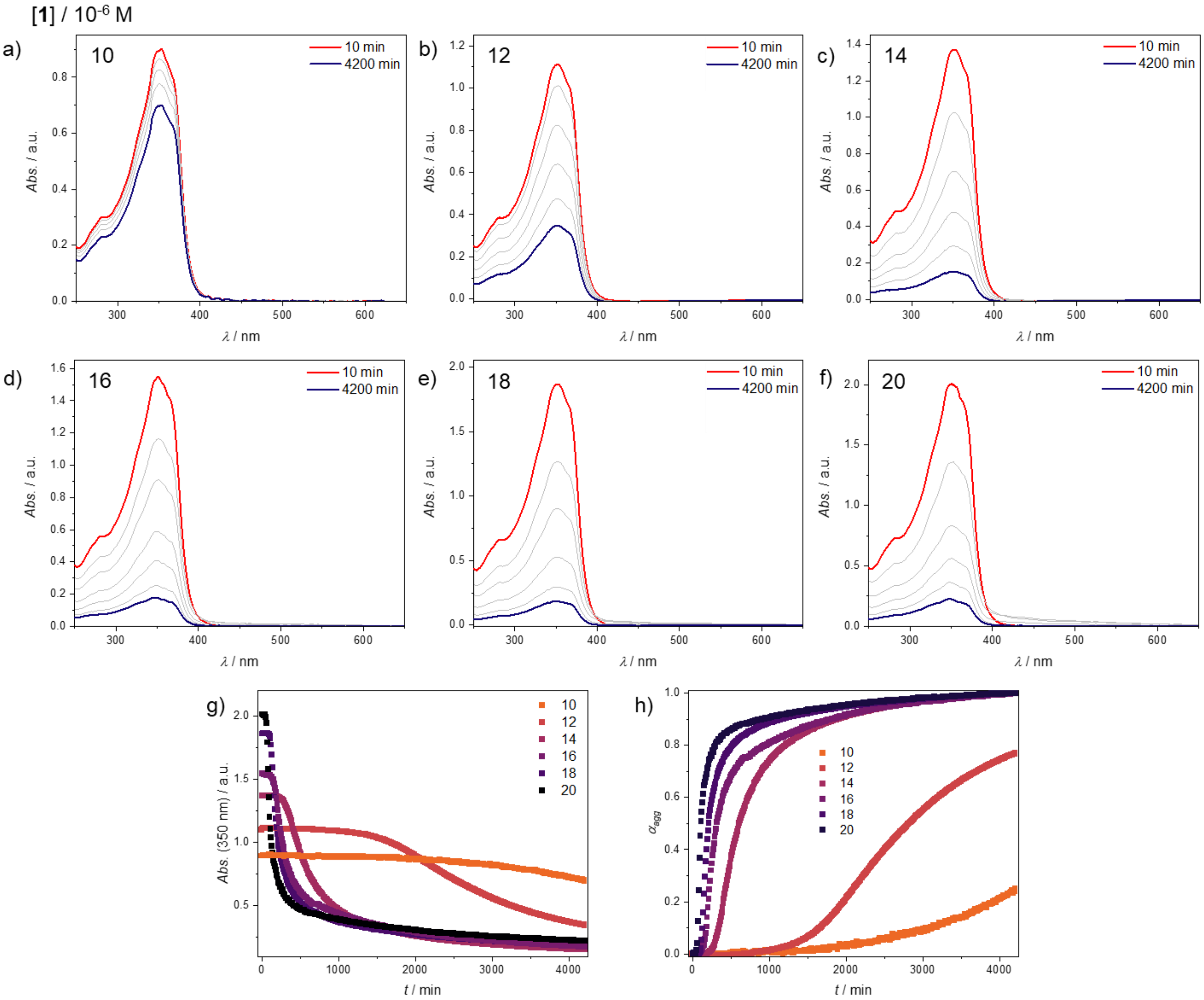

Supplementary Figure 4: a-f) Time-dependent UV/Vis spectra of **1** following the kinetic self-assembly protocol described in the method section at various concentrations (see insets for specific concentrations), with the time-dependent evolution of the absorbance at the absorption maximum (g; λ = 350 nm) and the resulting time-dependent aggregation curves (h) plotted below.

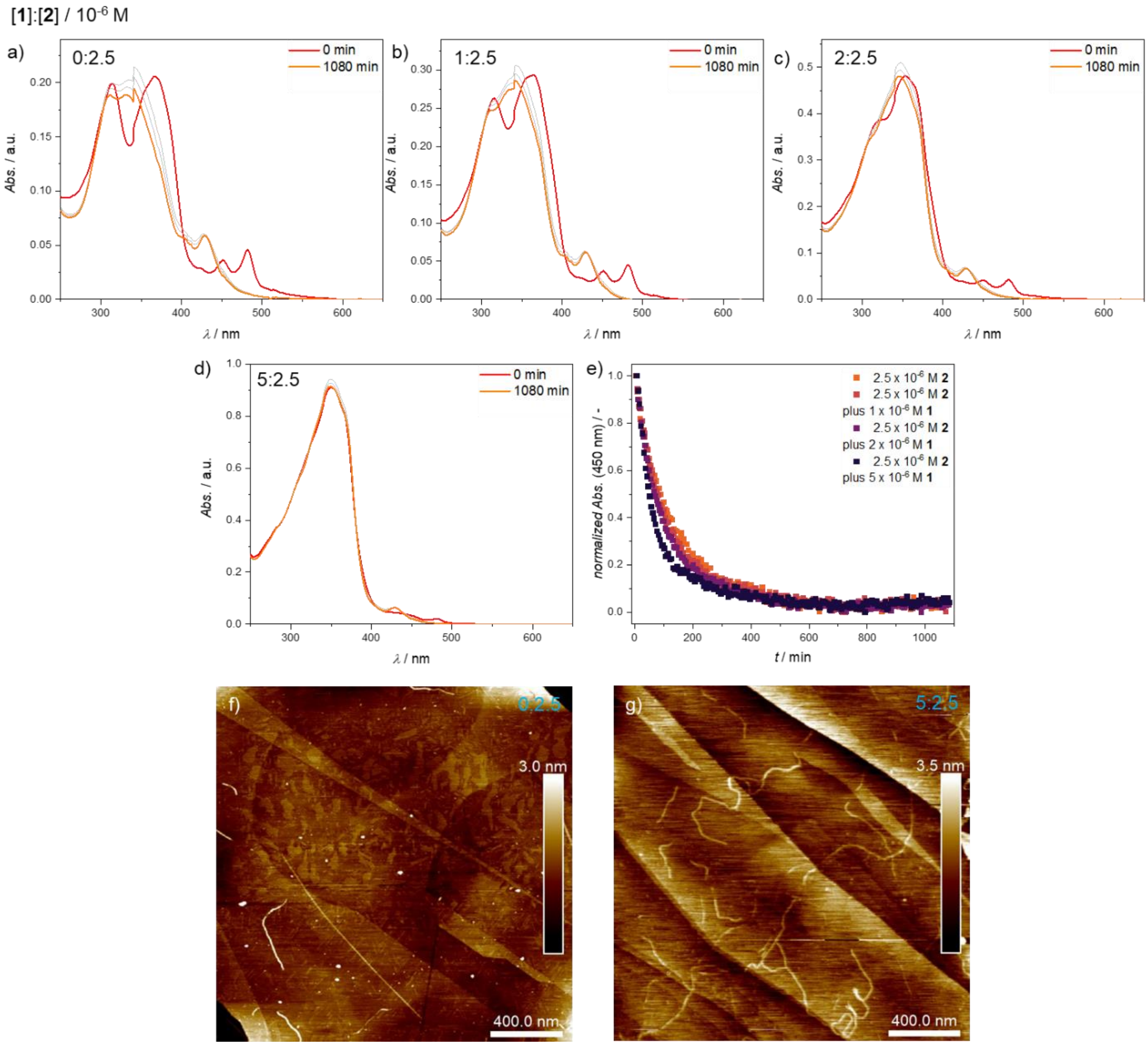

Supplementary Figure 5: Influence of **1** on the kinetic self-assembly behaviour of **2**. a-d) Time-dependent UV/Vis spectra of **1** and **2** at different ratios, where the concentration of **2** is kept constant. e) Time-dependent changes in absorbance at a characteristic wavelength for the aggregation of **2** (λ = 450 nm), using different amounts of added **1**. f + g) AFM height images displaying the unaffected morphology of Agg2 in the absence (f) and presence (g) of **1** under kinetic self-assembly conditions

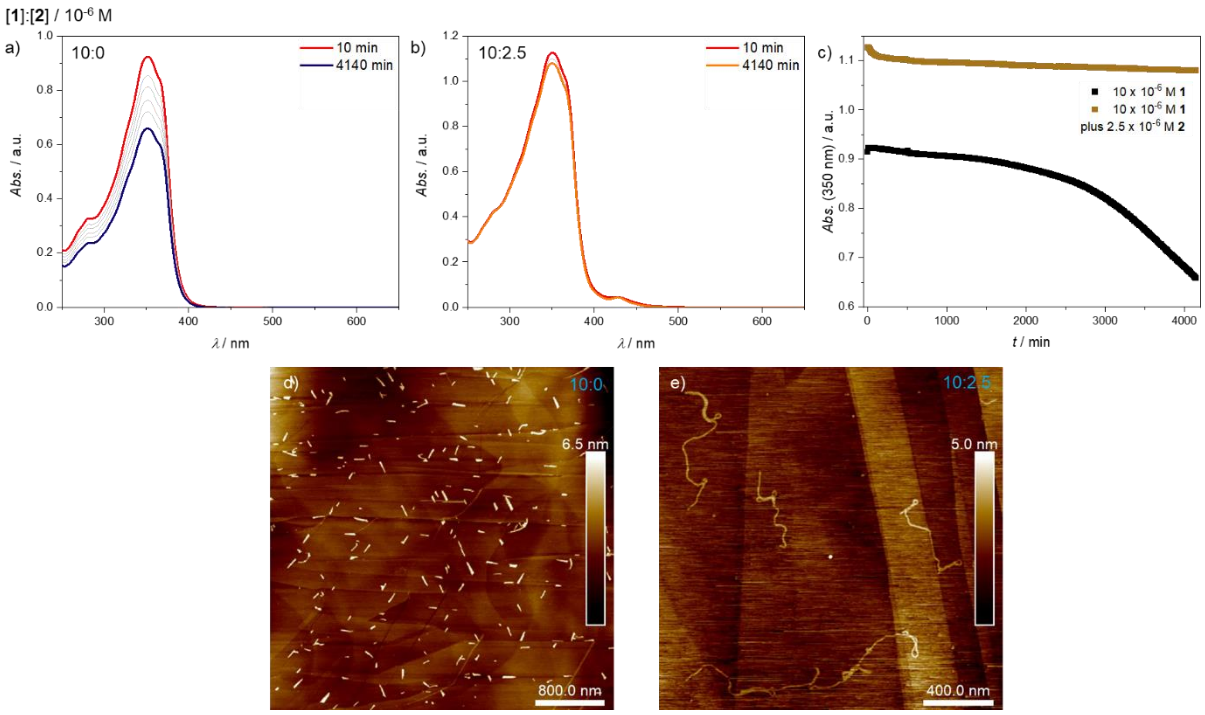

Supplementary Figure 6: a+b) Time-dependent UV/Vis spectra using a concentration of 10 x 10^{-6} M of **1** in isolation (a) and in the presence of 2.5 x 10^{-6} M of **2** (b), with the time-dependent changes in absorbance at the absorption maximum (c; λ = 350 nm). d+e) AFM height images recorded after the time-dependent UV/Vis studies, revealing Agg**1U** (d) and Agg**2** (e) depending on the crowding conditions.

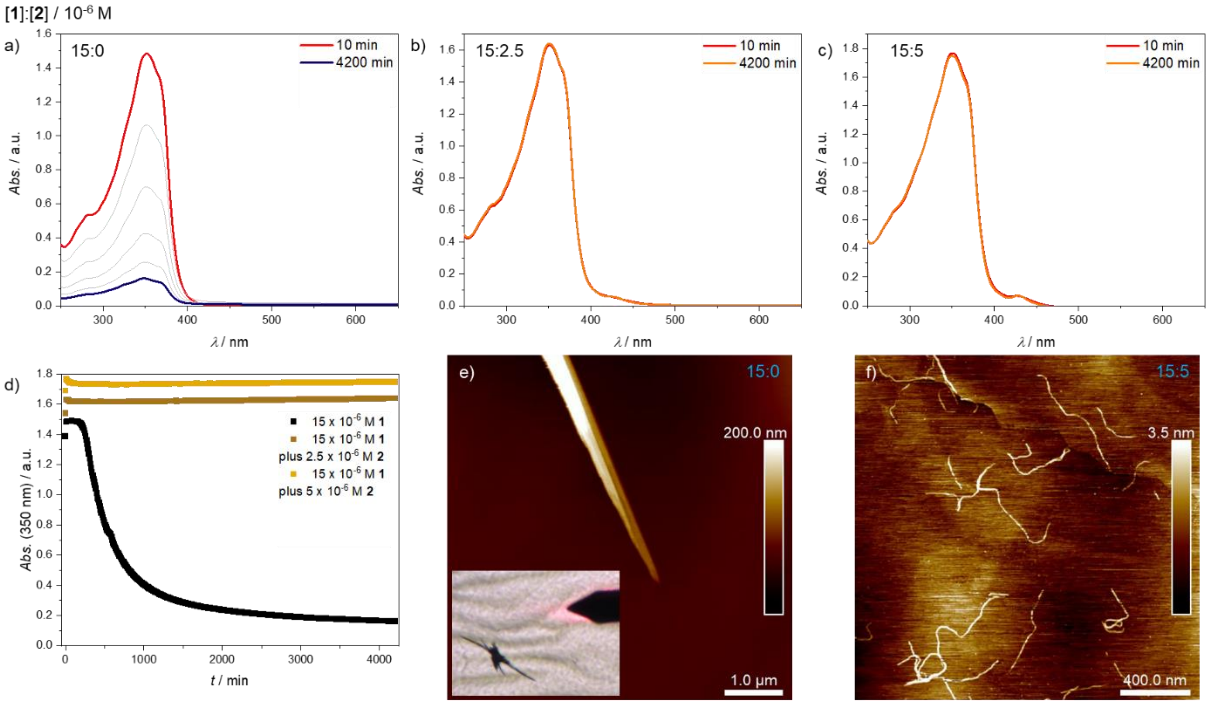

Supplementary Figure 7: a-c) Time-dependent UV/Vis spectra using a concentration of 15 x 10^{-6} M of **1** in isolation (a) and in the presence of 2.5 x 10^{-6} M (b) and 5 x 10^{-6} M of **2** (c). d) Time-dependent changes in absorbance at the absorption maximum (λ = 350 nm). e+f) AFM height images recorded after the time-dependent UV/Vis studies, revealing Agg**1U** (e) and Agg**2** (f) depending on the crowding conditions.

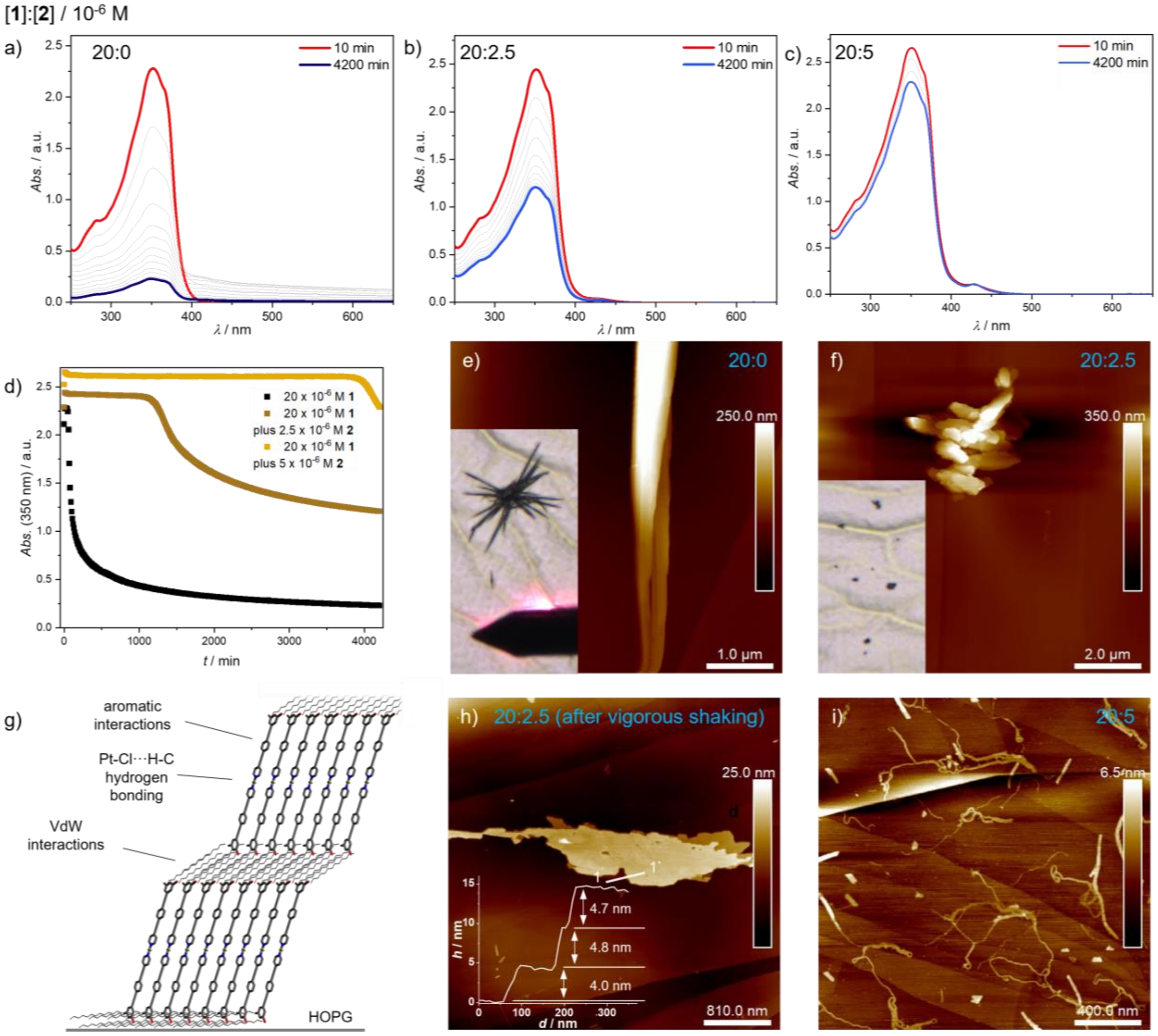

Supplementary Figure 8: a-c) Time-dependent UV/Vis spectra using a concentration of 20 × 10^{-6} M of **1** in isolation (a) and in the presence of 2.5 × 10^{-6} M (b) and 5 × 10^{-6} M of **2** (c), with the time-dependent absorbance at the absorption maximum (d; λ = 350 nm). e-g) AFM height images recorded after the time-dependent UV/Vis studies, revealing Agg**1U** (e), Agg**1C** (f+h) and Agg**2** (i) depending on the crowding conditions. The inset in (h) corresponds to the height profile along 1-1` in (h). g) schematic depiction of the supramolecular packing of **1** in Agg**1C**.

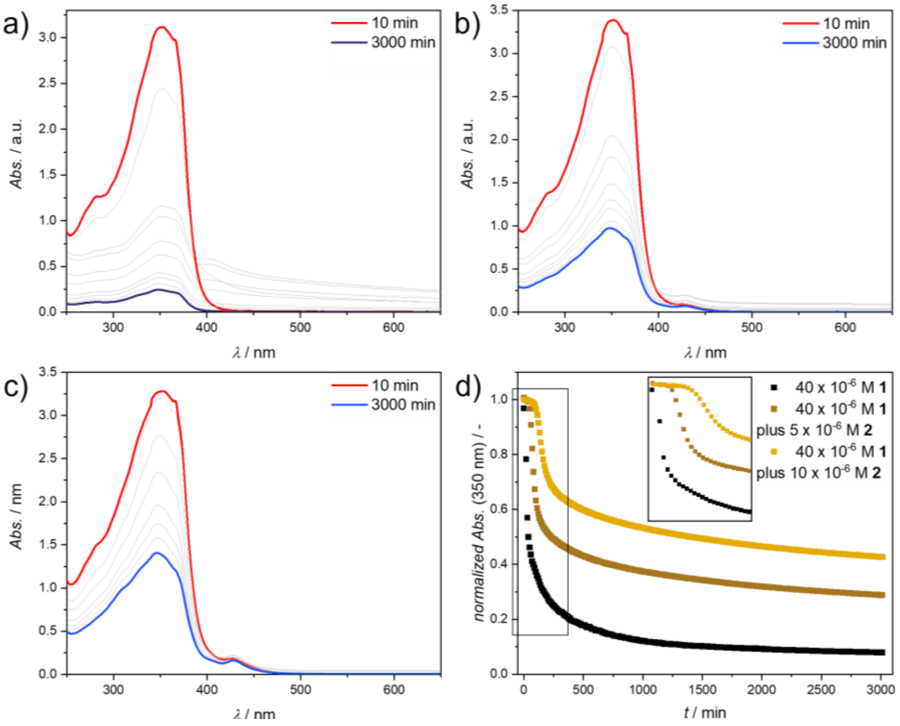

Supplementary Figure 9: a-d) Time-dependent UV/Vis spectra of mixtures of **1** and **2** following the kinetic self-assembly protocol described in the method section at 40 x 10^{-6} M of **1** in the presence of increasing amounts of **2** (c_2 = 0 x 10^{-6} M (a); c_2 = 5 x 10^{-6} M (b); c_2 = 10 x 10^{-6} M (c)) with the absorbance at the absorption maximum (λ = 350 nm) plotted against the time (d).

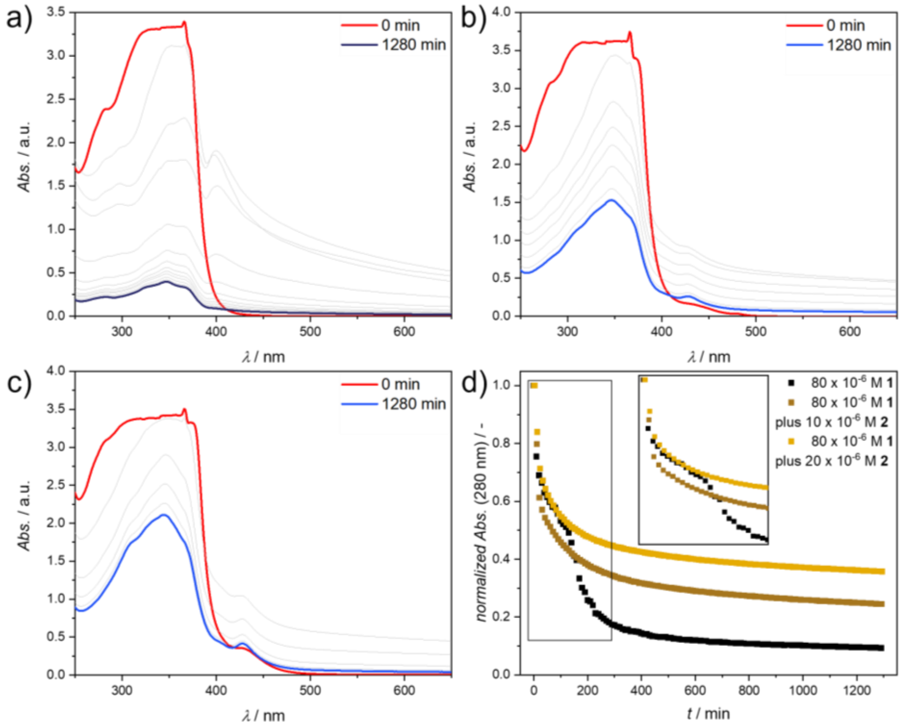

Supplementary Figure 10: a-d) Time-dependent UV/Vis spectra of mixtures of **1** and **2** following the kinetic self-assembly protocol described in the method section at 80 x 10^{-6} M of **1** in the presence of increasing amounts of **2** (c_2 = 0 x 10^{-6} M (a); c_2 = 10 x 10^{-6} M (b); c_2 = 20 x 10^{-6} M (c)) with the absorbance at λ = 280 nm plotted against the time (d).

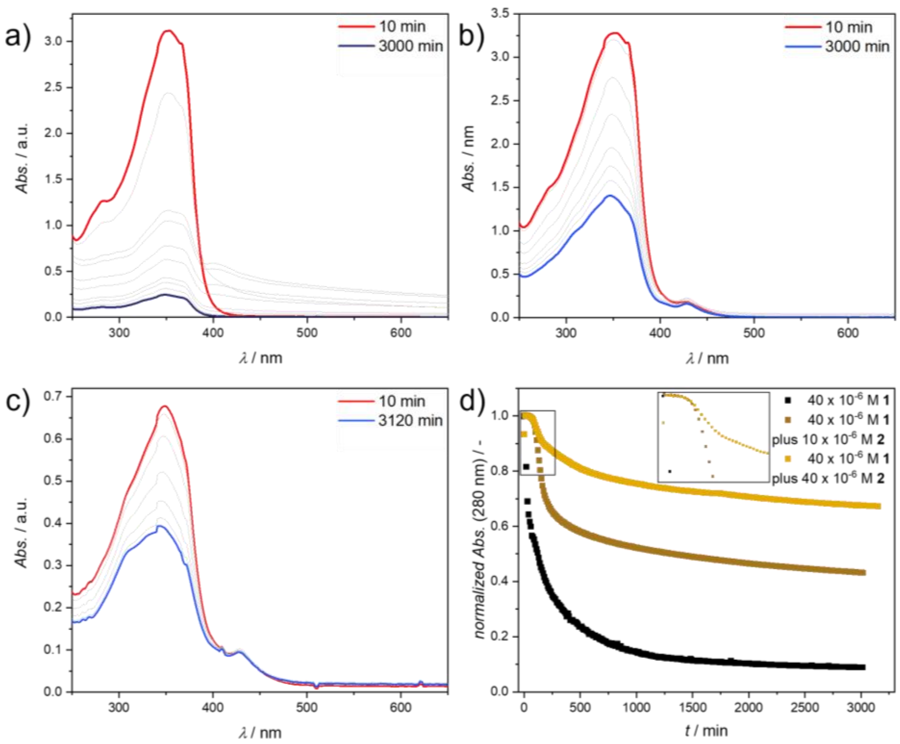

Supplementary Figure 11: a-d) Time-dependent UV/Vis spectra of mixtures of **1** and **2** following the kinetic self-assembly protocol described in the method section at 40 x 10^{-6} M of **1** in the presence of increasing amounts of **2** (c_2 = 0 x 10^{-6} M (a); c_2 = 10 x 10^{-6} M (b); c_2 = 40 x 10^{-6} M (c)) with the absorbance at λ = 350 nm plotted against the time (d).

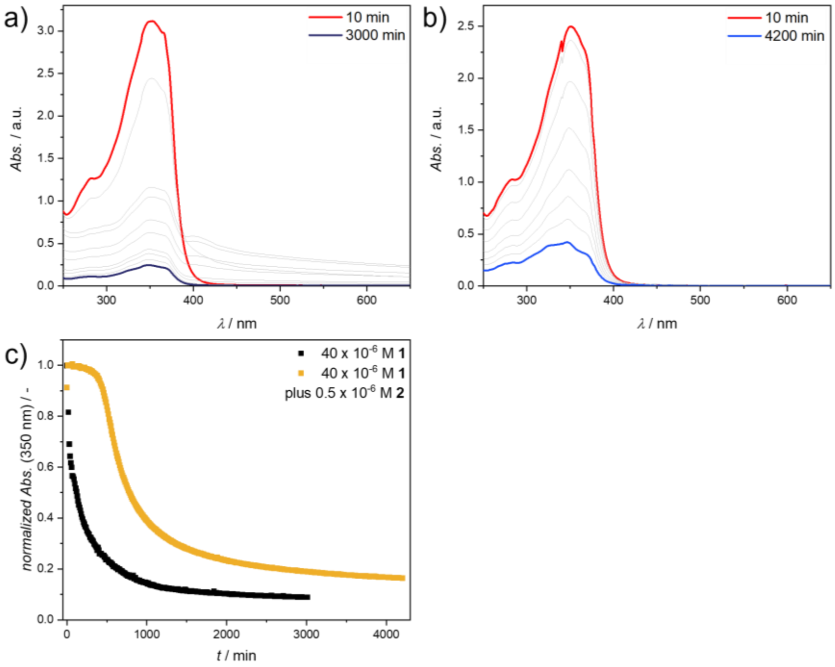

Supplementary Figure 12: a-c) Time-dependent UV/Vis spectra of mixtures of **1** and **2** following the kinetic self-assembly protocol described in the method section at 40 x 10^{-6} M of **1** in the presence of increasing amounts of **2** (c_2 = 0 x 10^{-6} M (a); c_2 = 0.5 x 10^{-6} M (b)) with the absorbance at the absorption maximum (λ = 350 nm) plotted against the time (c).

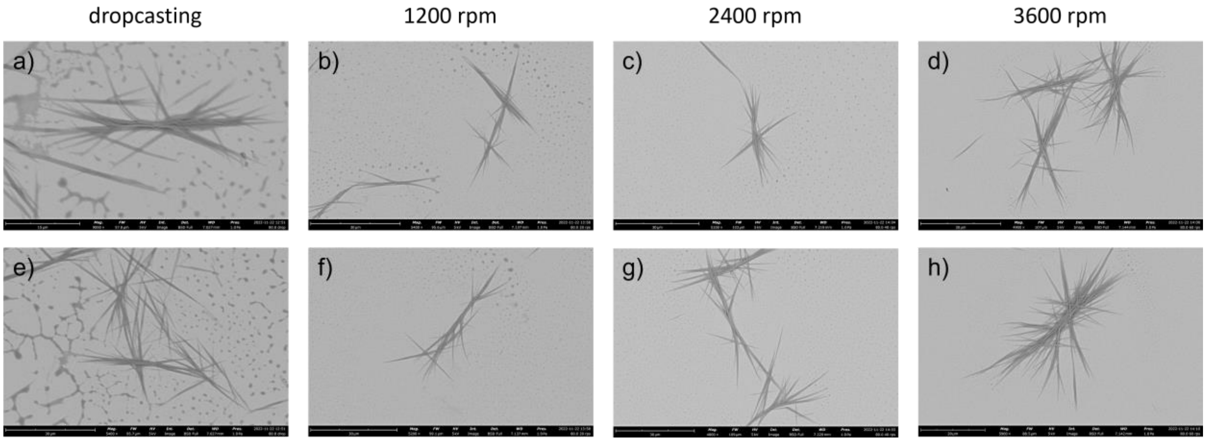

Supplementary Figure 13: a-h) Spin rate-dependent SEM analysis of Agg**1U** using a concentration of c_1 = 80 x 10^{-6} M, revealing the stability of Agg**1U** against different drying processes. Samples were prepared using dropcasting (a,e); 1200 rpm (d,f), 2400 rpm (c,g) and 3600 rpm (d,h).

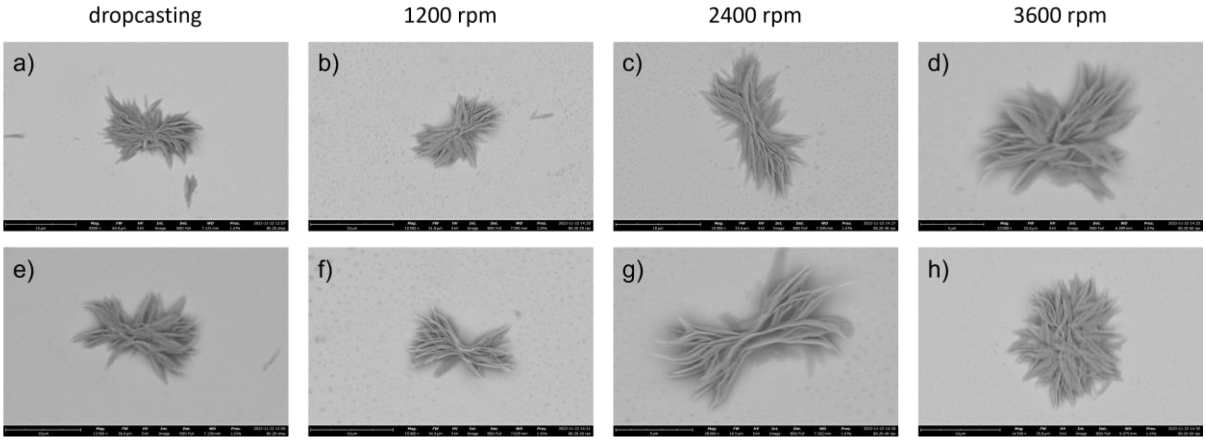

Supplementary Figure 14: a-h) Spin rate-dependent SEM analysis of Agg**1C** using a concentration of c_1 = 80 x 10^{-6} M; c_2 = 20 x 10^{-6} M, revealing the stability of Agg**1C** against different drying processes. Samples were prepared using dropcasting (a,e); 1200 rpm (d,f), 2400 rpm (c,g) and 3600 rpm (d,h).

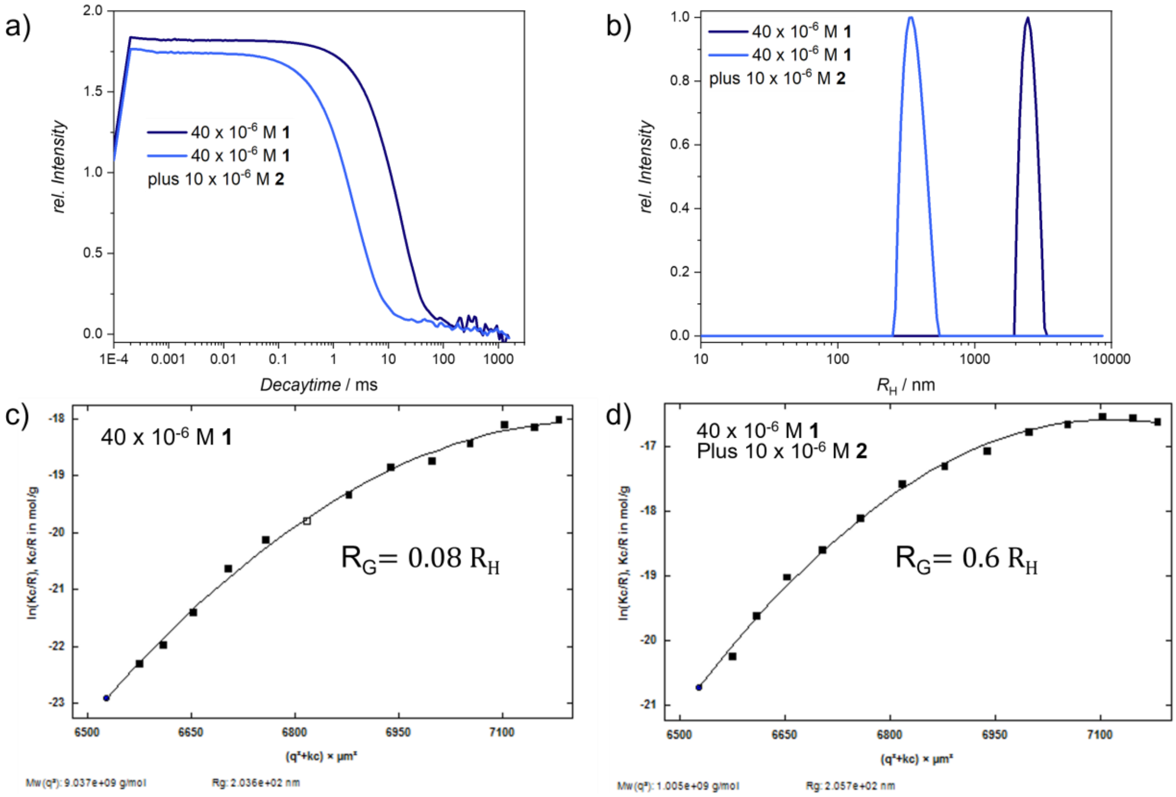

Supplementary Figure 15: a-d) Results from DLS (a+b) and SLS (c+d) studies of Agg**1U** and Agg**1C**. a) DLS correlation function of Agg**1** in uncrowded and crowded conditions. b) DLS size distribution of Agg**1** in uncrowded and crowded conditions. Guinier plot of SLS studies of Agg**1U** (c) and Agg**1C** (d).

Analysis of self-sorted self-assembly of 1 and 2 under kinetic conditions

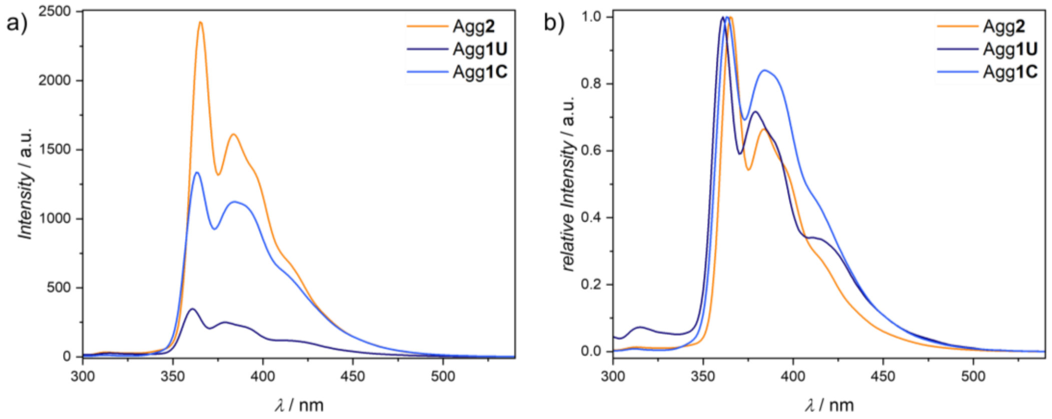

Supplementary Figure 16: Photoluminescence (a) and normalized photoluminescence (b) spectra of Agg**2**, Agg**1U** and Agg**1C** at c_2 = 10 x 10^{-6} M (Agg**2**), c_1 = 40 x 10^{-6} M (Agg**1U**) and c_2 = 10 x 10^{-6} M with c_1 = 40 x 10^{-6} M (**1**, Agg**1C**).

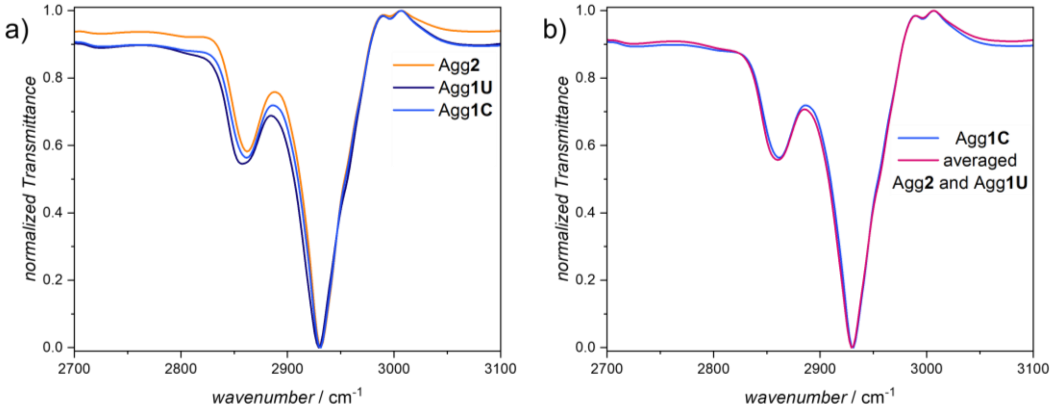

Supplementary Figure 17: a) FT-IR spectra of Agg**2**, Agg**1U** and Agg**1C** in thin film. b) comparison between the FT-IR spectra of Agg**1C** and the averaged spectra of Agg**2** and Agg**1U** according to the ratio in the thin film of Agg**1C** (1:4).

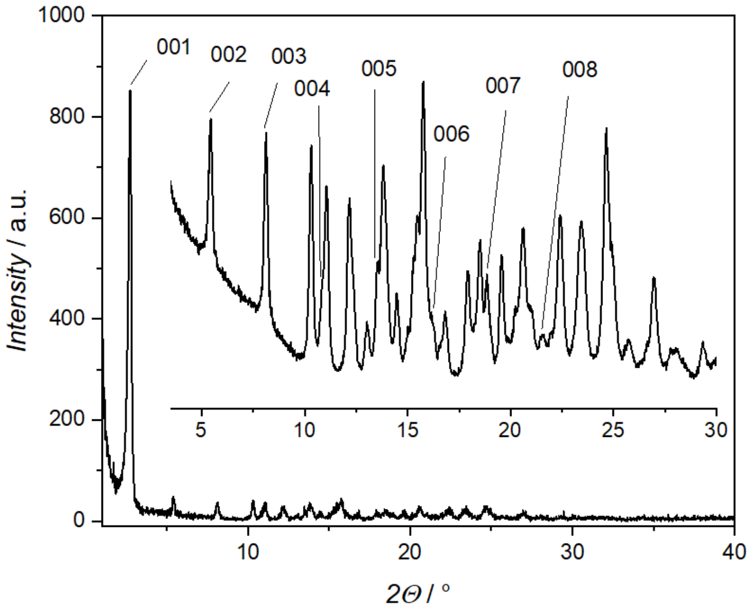

Supplementary Figure 18: XRD patterns of Agg**1U** between 2 and 40° with the insets depicting the range between 3 and 30°.

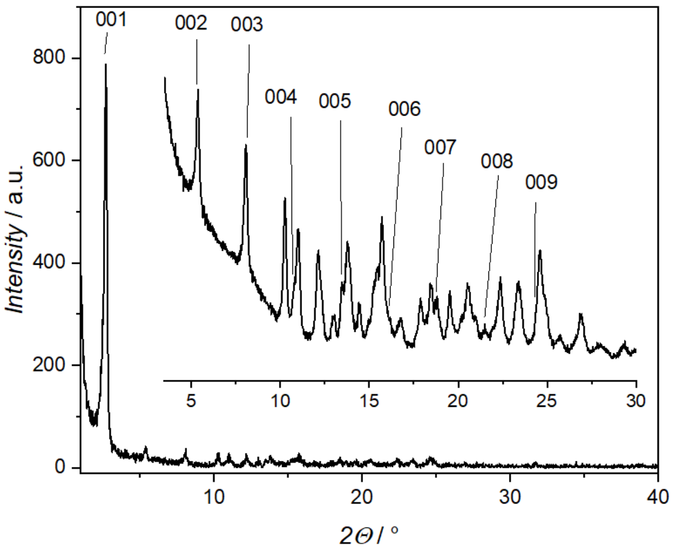

Supplementary Figure 19: XRD patterns of Agg**1C** between 2 and 40° with the insets depicting the range between 3 and 30°.

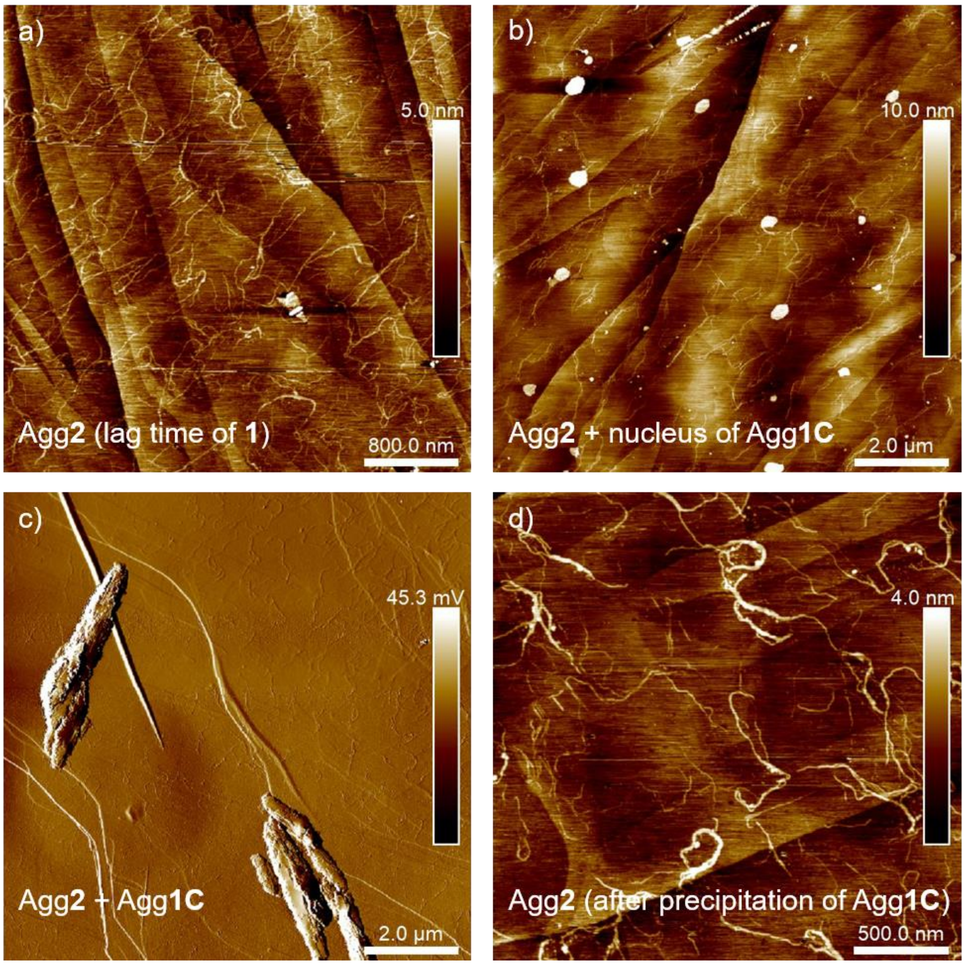

Supplementary Figure 20: AFM images revealing the narcissistic self-sorting of **1** and **2** over time, following the kinetic self-assembly protocol described in the methods section. a, AFM height image of Agg**2** obtained shortly after the thermal quenching. b, AFM height image revealing small plates of Agg**1C** together with Agg**2** obtained shortly after the self-assembly of **1** started. c, representative AFM phase image of Agg**1C** and Agg**2** obtained after a short equilibration period (prior to precipitation of large flower-like structures of Agg**1C**). d, AFM height image of Agg**2** obtained after full equilibration (precipitation of the flower-like structures of Agg**1C**). $c_1 = 40 \times 10^{-6}$ M; $c_2 = 5 \times 10^{-6}$ M.

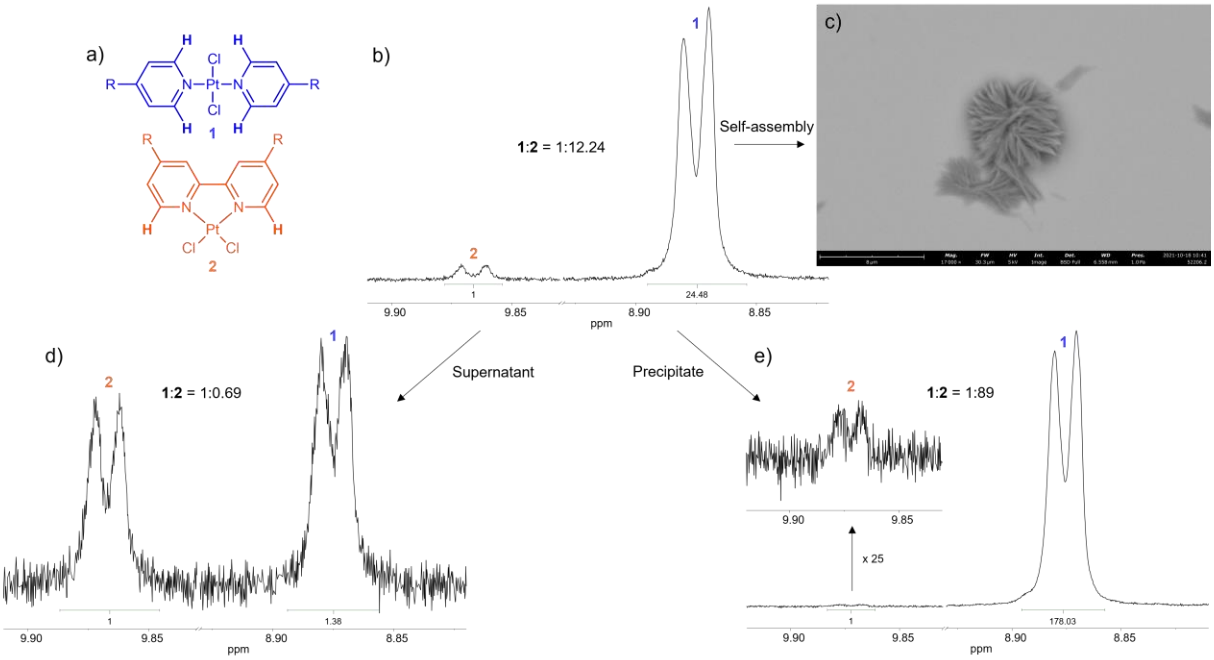

Supplementary Figure 21: a) Chemical structure of **1** and **2** highlighting the relevant protons depicted in the NMR spectra; b) ^{1}H NMR spectra of a mixture of **1** and **2** in chloroform, with an SEM micrograph obtained after conducting the kinetic self-assembly protocol (c). d+e) ^{1}H NMR spectra of the resulting supernatant (d) and precipitate (e), highlighting the narcissistic self-sorting in Agg**1C**.

Insights into the mechanism of supramolecular crowding in binary mixtures of 1 and Agg2

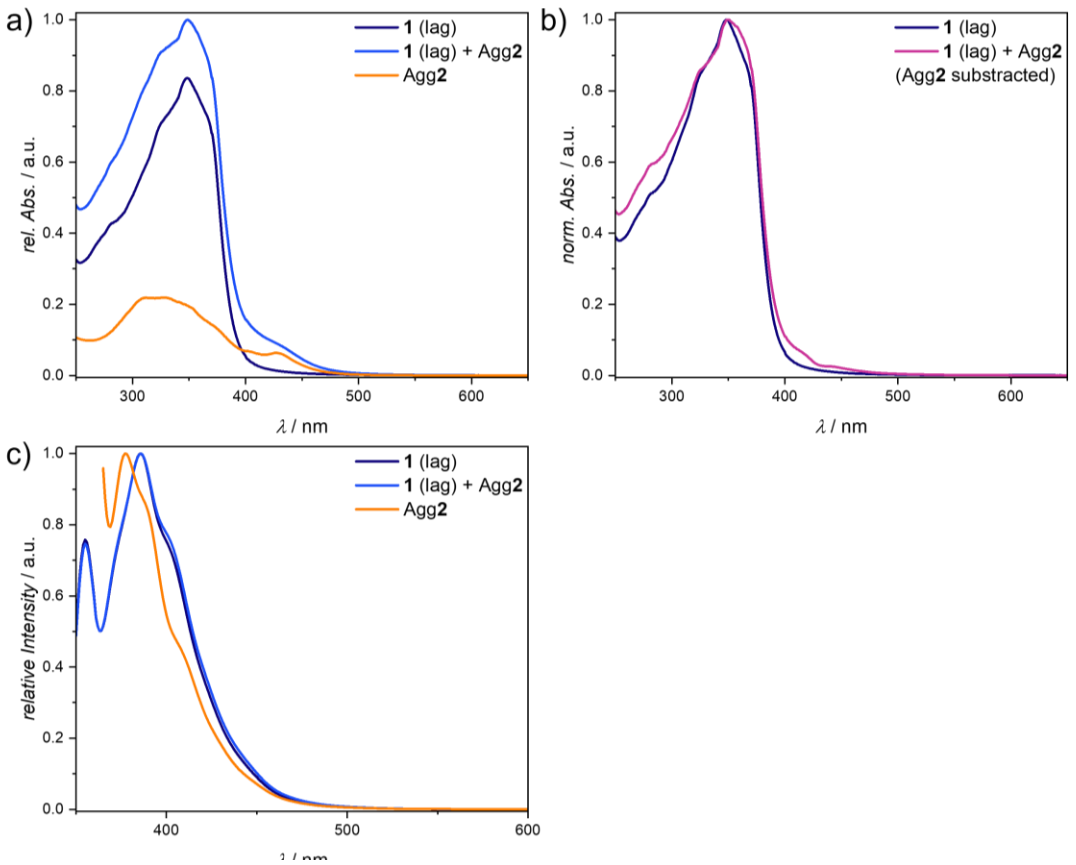

Supplementary Figure 22: a+b) UV/Vis spectra of **1** within the lag time in the absence and presence of Agg**2**, with the UV/Vis spectrum of Agg**2** shown for comparison (a) and the normalized UV/Vis spectra of **1** in the absence and presence of Agg**2** (b). c) Photoluminescence spectra of **1** in the absence and presence of Agg**2** with the spectrum of Agg**2** shown for comparison. (c_1 = 20 x 10^{-6} M; c_2 = 5 x 10^{-6} M; $\lambda_{Ex.}$ = 340 nm).

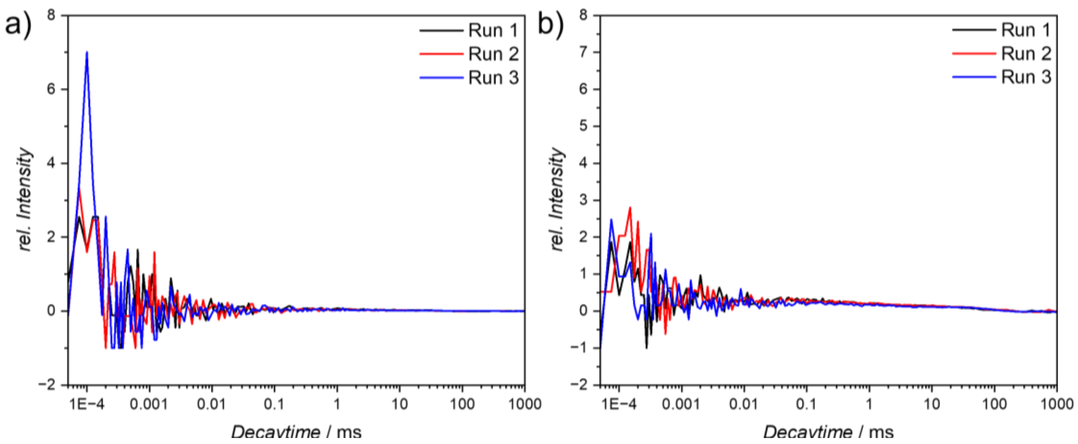

Supplementary Figure 23: a,b) DLS correlation function of Agg**2** using a concentration of 5 x 10^{-6} M in isolation (a) and in the presence of **1** (b, c_1 = 20 x 10^{-6} M).

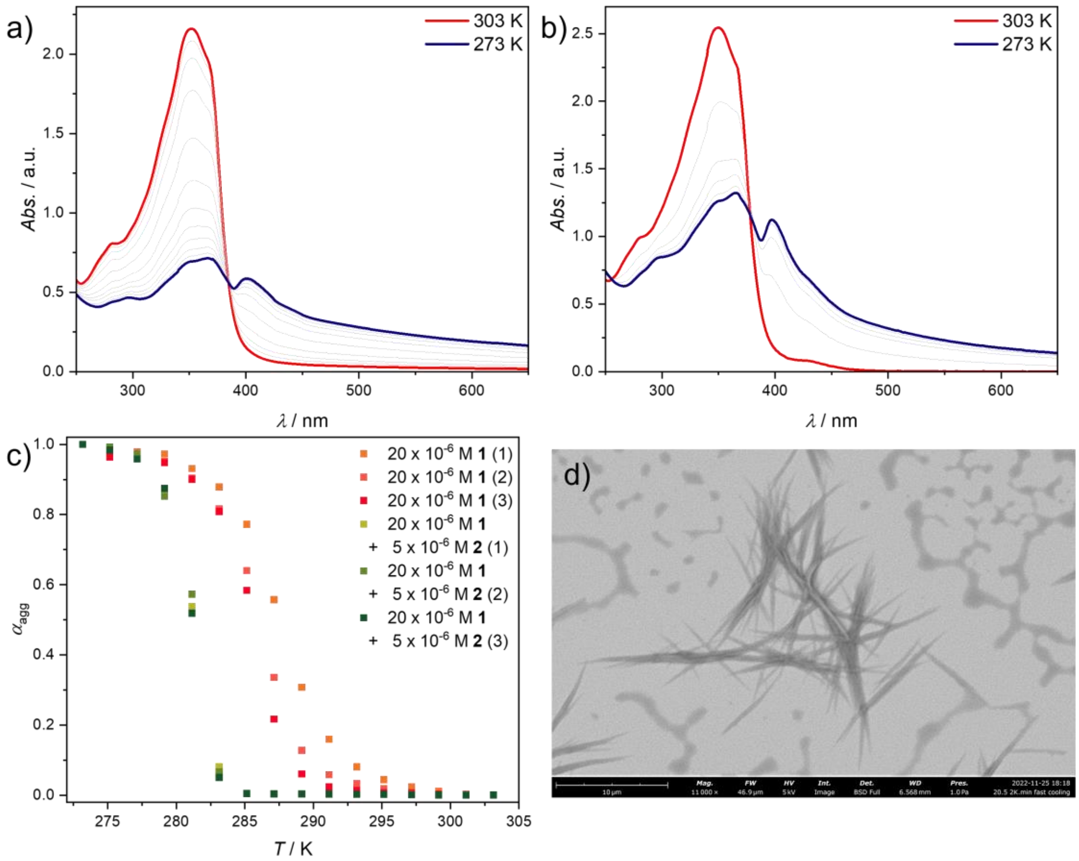

Supplementary Figure 24: a+b) VT UV/Vis spectra of **1** in the absence (a, c_1 = 20 x 10^{-6} M) and in the presence of Agg**2** (b, c_1 = 20 x 10^{-6} M; c_2 = 5 x 10^{-6} M) with the changes in α_{agg} plotted against the temperature (c) using fast cooling rates (2 K/min and 2 K data interval) for multiple repeated measurements. d) SEM micrograph of Agg**1U** formed in the presence of Agg**2** under fast cooling conditions.

Evaluation of the thermodynamic stability of Agg1U and Agg1C

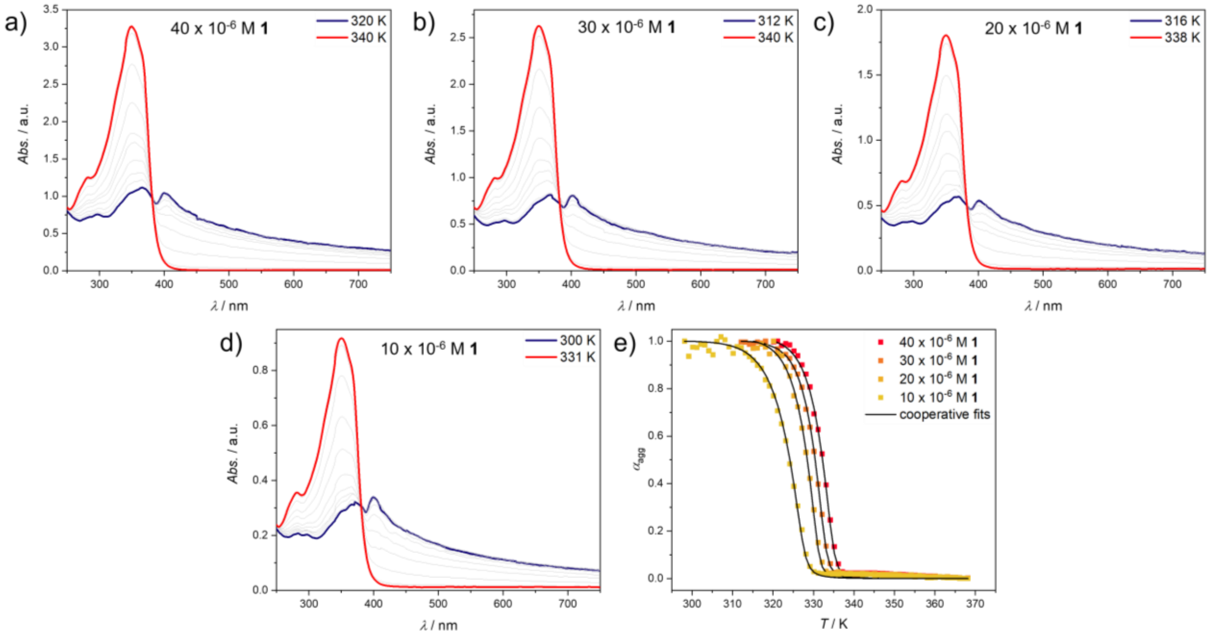

Supplementary Figure 25: a-d) Temperature-dependent UV/Vis spectra of Agg**1U** following the heating protocol described in the method section, using c_1 = 40 x 10^{-6} M (a); c_1 = 30 x 10^{-6} M (b); c_1 = 20 x 10^{-6} M (c); c_1 = 10 x 10^{-6} M (d) with the corresponding aggregation curves and individual cooperative fits based on the nucleation-elongation model shown in (e).

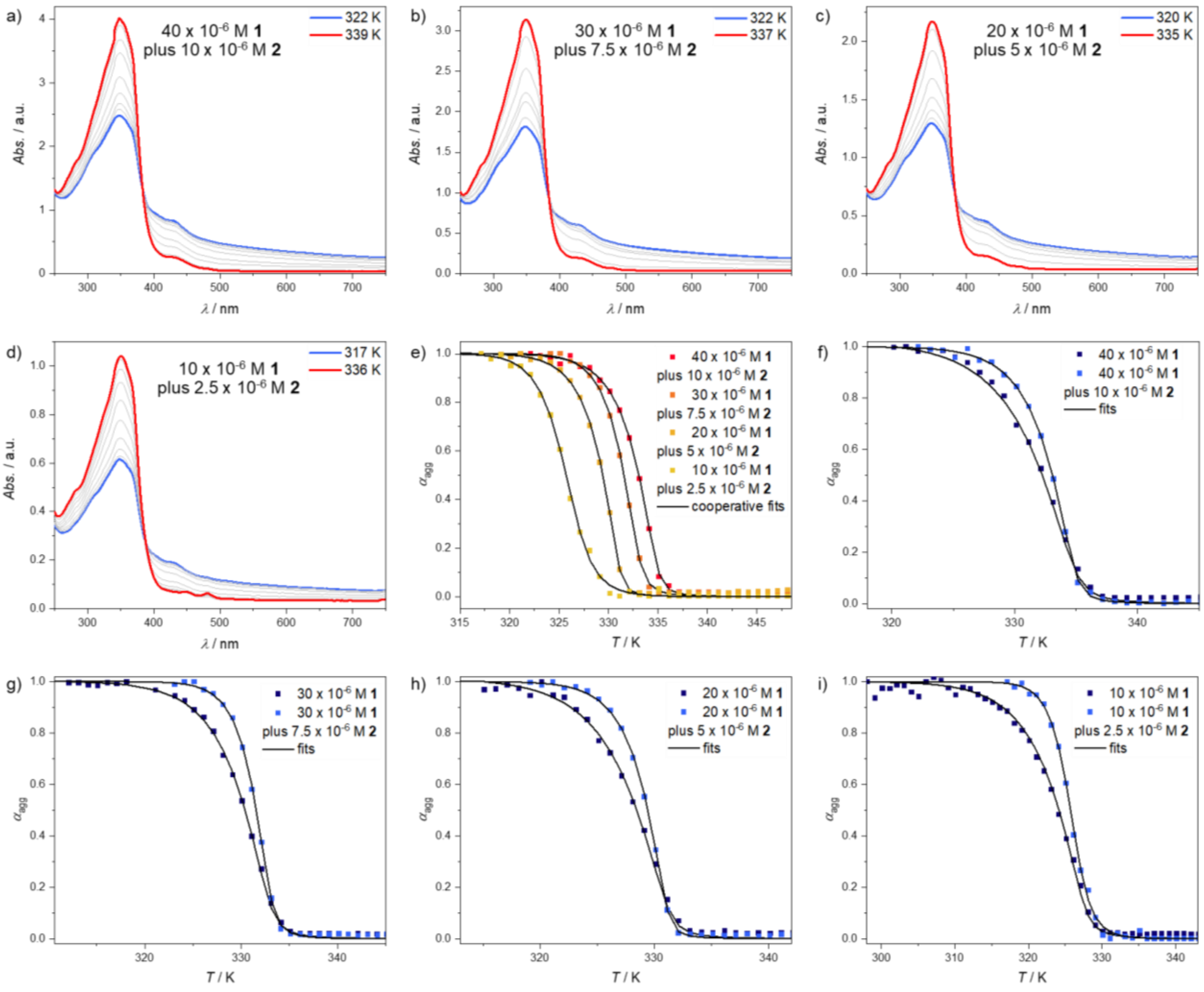

Supplementary Figure 26**:** a-d) Temperature-dependent UV/Vis spectra of Agg**1C** following the heating protocol described in the method section, using a range of different concentrations (see insets for specific concentrations). e) The corresponding aggregation curves and individual cooperative fits based on the nucleation-elongation model. f-i) Visual comparison between the aggregation curves of Agg**1U** and Agg**1C** obtained under heating conditions for various concentrations revealing an increase in the hierarchical stability of Agg**1C**, while the nucleation regime remains largely unaffected.

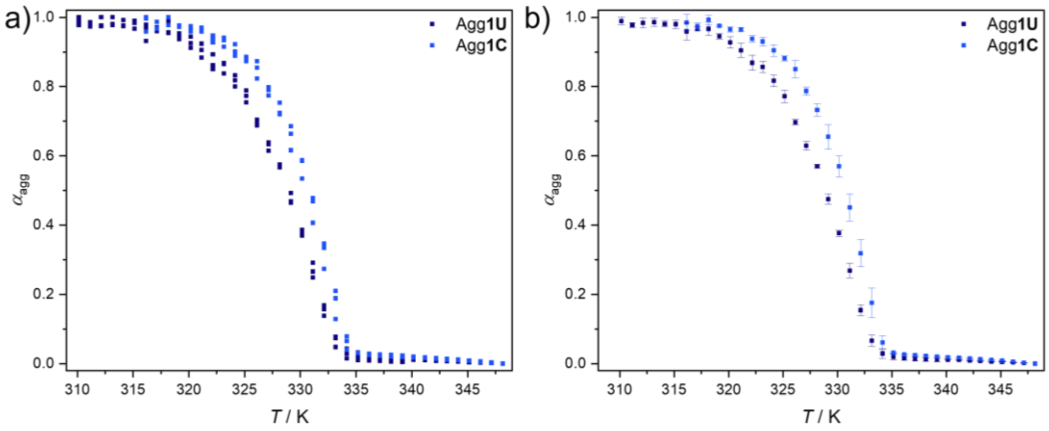

Supplementary Figure 27: Reproducibility study of the observed apparent increase in hierarchical stability using a concentration of $c_1 = 20 \times 10^{-6}$ M (Agg**1U**) and $c_1 = 20 \times 10^{-6}$ M; $c_2 = 5 \times 10^{-6}$ M (Agg**1C**). The graphs depict individual studies (a) and the average values with error bars depicting the first standard deviation (b).

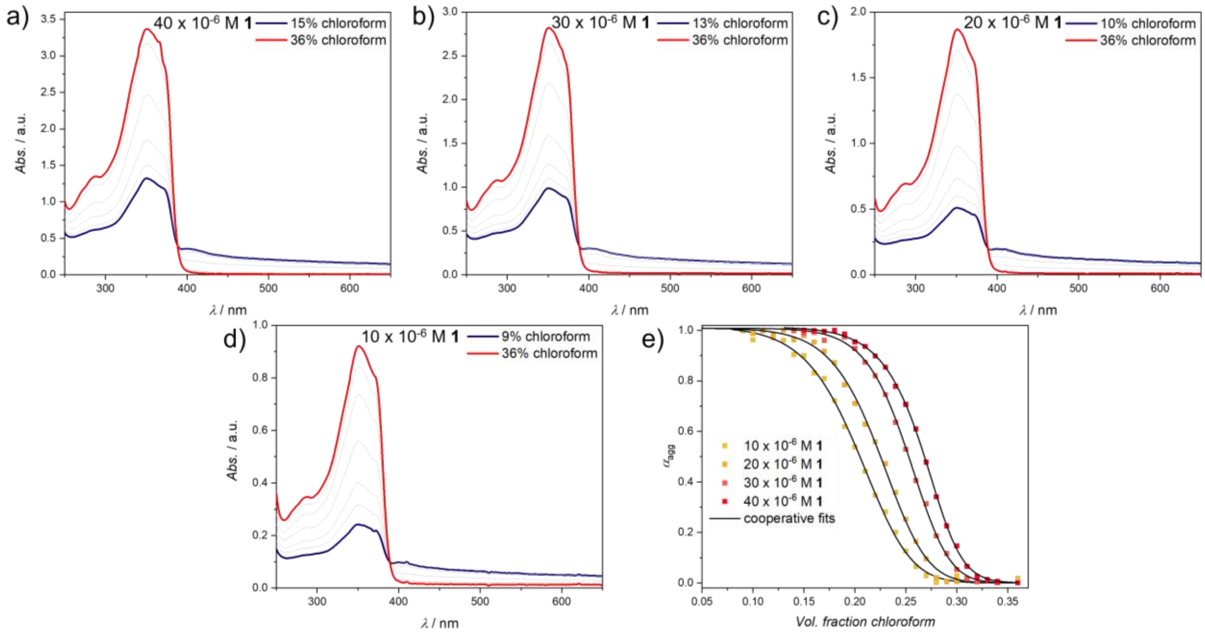

Supplementary Figure 28: a-d) Solvent-dependent UV/Vis spectra of Agg**1U** following the denaturation protocol described in the method section, using $c_{\mathbf{1}}$ = 40 x 10^{-6} M (a); $c_{\mathbf{1}}$ = 30 x 10^{-6} M (b); $c_{\mathbf{1}}$ = 20 x 10^{-6} M (c); $c_{\mathbf{1}}$ = 10 x 10^{-6} M (d) with the corresponding aggregation curves and cooperative fits based on the denaturation model shown in (e). Fits were obtained using a global fitting approach.

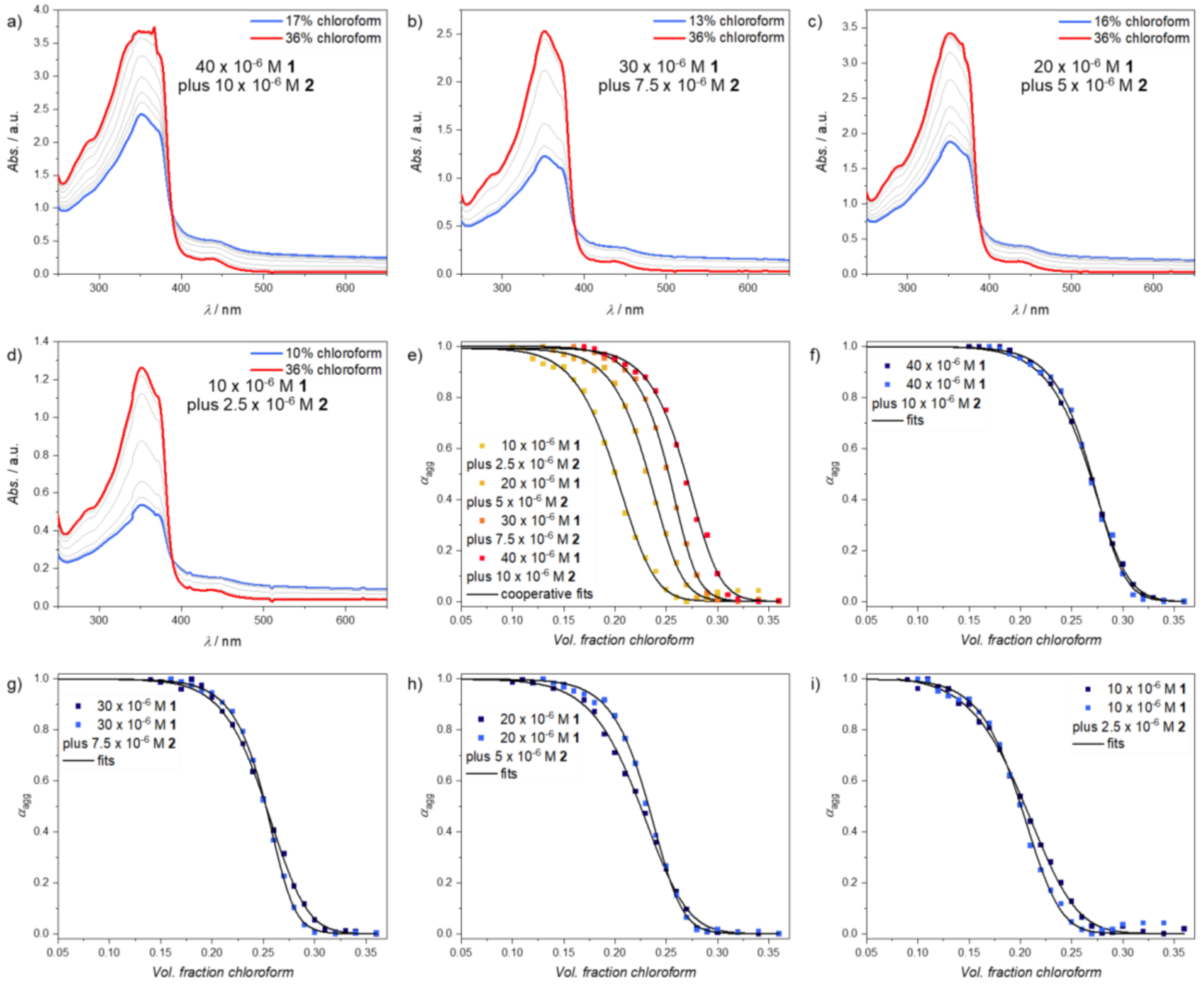

Supplementary Figure 29: a-d) Solvent-dependent UV/Vis spectra of Agg**1C** following the denaturation protocol described in the method section, using a range of different concentrations (see insets for specific concentrations). e) The corresponding aggregation curves and cooperative fits based on the denaturation model. Fits were obtained using a global fitting approach. f-i) Visual comparison between the aggregation curves of Agg**1U** and Agg**1C** obtained under denaturation conditions for various different concentrations revealing a nearly identical behaviour in denaturation experiments.

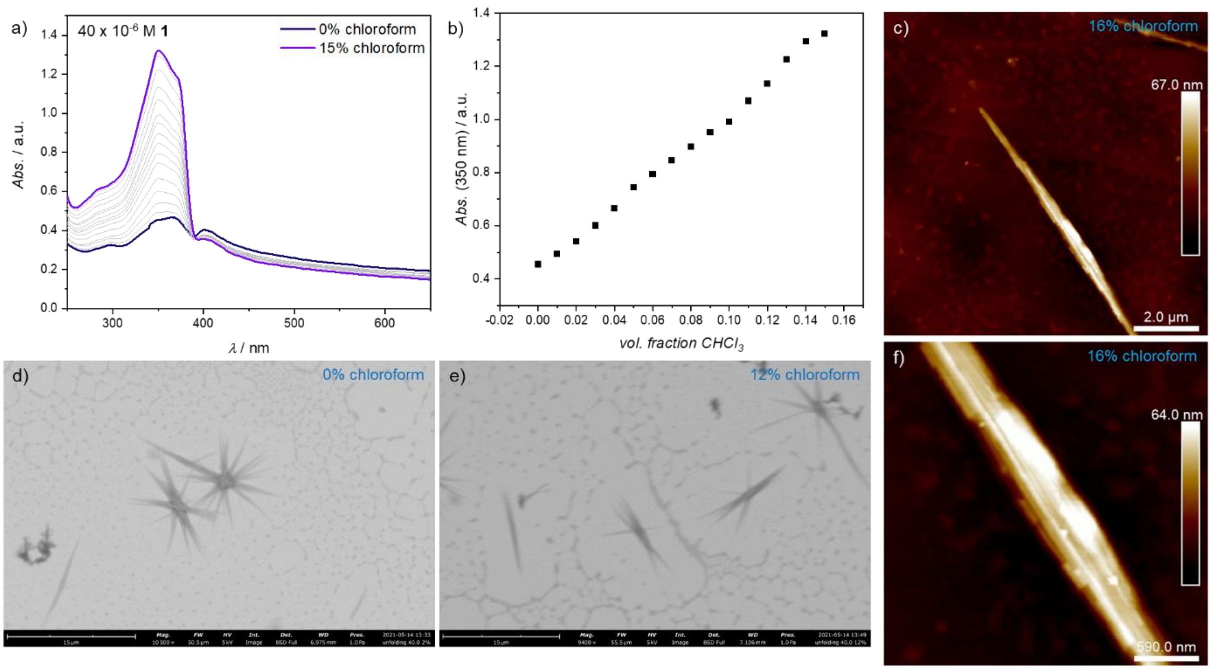

Supplementary Figure 30: a+b) Solvent-dependent UV/Vis spectra of Agg**1U** in low polarity solvent mixtures (a) with the absorbance at the absorption maximum (λ = 350 nm) plotted against the volume fraction of chloroform revealing a linear relationship (b). c-f) SEM micrographs at low contents of chloroform (d+e), with AFM height images on the right revealing a partial debundling of the one-dimensional fibres (c+f).

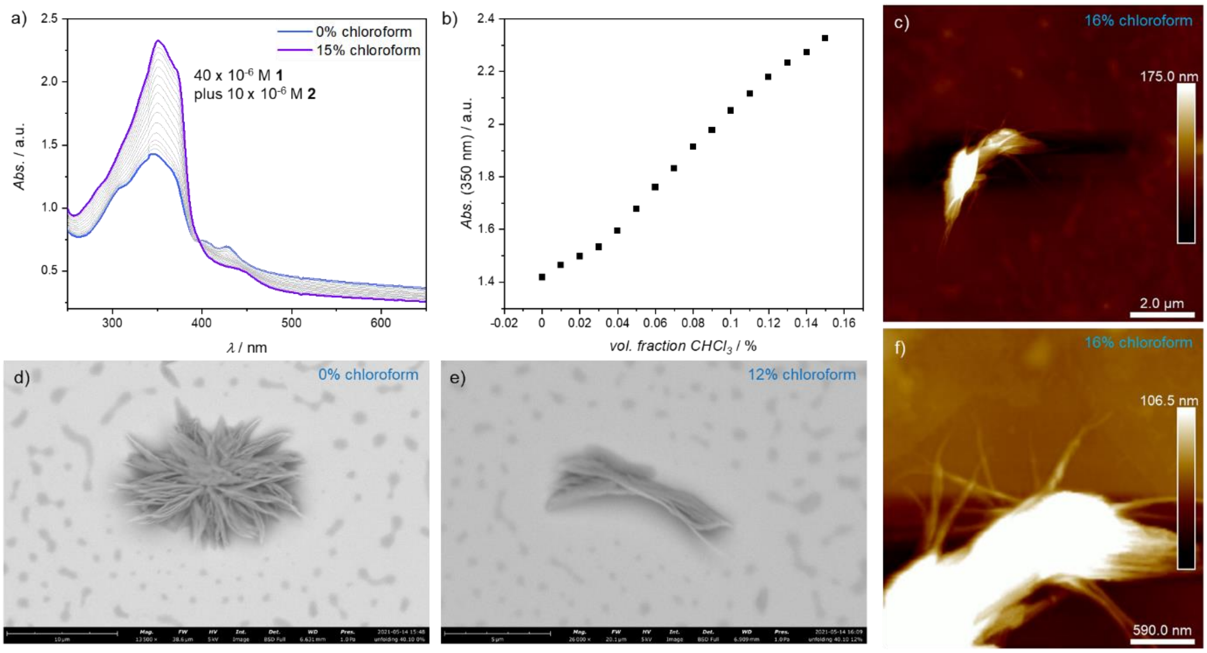

Supplementary Figure 31: a+b) Solvent-dependent UV/Vis spectra of Agg**1C** in low polarity solvent mixtures (a) with the absorbance at the absorption maximum (λ = 350 nm) plotted against the volume fraction of chloroform revealing a linear relationship (b). c-f) SEM micrographs at low contents of chloroform (d+e), with AFM height images on the right revealing a partial debundling of the two-dimensional plates into one-dimensional lamellae (c+f).

Time-dependent morphological transformation of Agg1C

72 h [**1**]:[**2**] 10^{-6} M

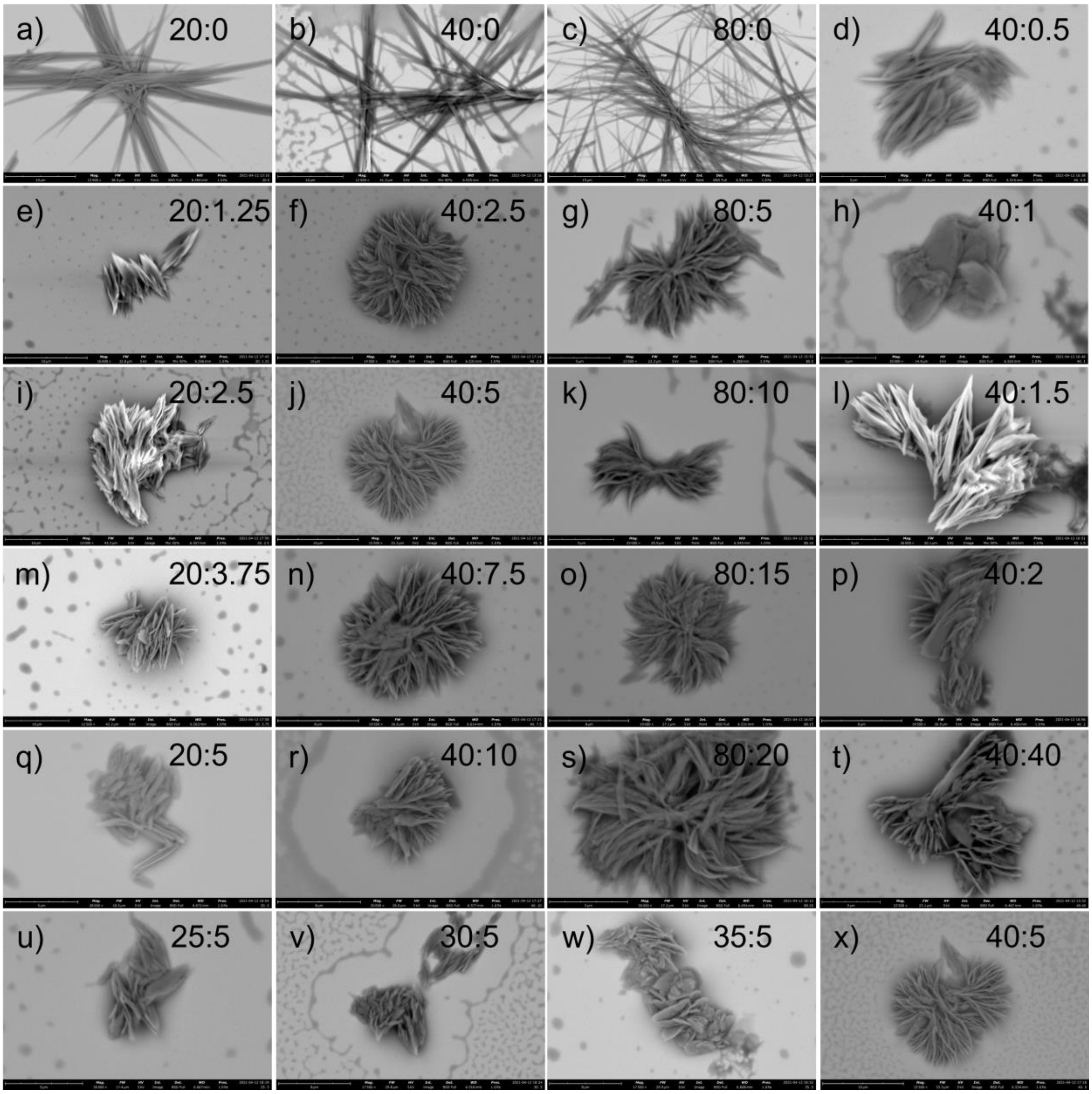

Supplementary Figure 32: a-x) SEM micrographs of Agg**1** three days after applying the kinetic self-assembly protocol described in the extended information; the specific ratio between **1** and **2** used to investigate the morphology upon crowding were: c_1 = 20 x 10^{-6} M; c_2 = 0 x 10^{-6} M (a); c_1 = 40 x 10^{-6} M; c_2 = 0 x 10^{-6} M (b); c_1 = 80 x 10^{-6} M; c_2 = 0 x 10^{-6} M (c); c_1 = 40 x 10^{-6} M; c_2 = 0.5 x 10^{-6} M (d); c_1 = 20 x 10^{-6} M; c_2 = 1.25 x 10^{-6} M (e); c_1 = 40 x 10^{-6} M; c_2 = 2.5 x 10^{-6} M (f); c_1 = 80 x 10^{-6} M; c_2 = 5 x 10^{-6} M (g); c_1 = 40 x 10^{-6} M; c_2 = 1 x 10^{-6} M (h); c_1 = 20 x 10^{-6} M; c_2 = 2.5 x 10^{-6} M (i); c_1 = 40 x 10^{-6} M; c_2 = 5 x 10^{-6} M (j); c_1 = 80 x 10^{-6} M; c_2 = 10 x 10^{-6} M (k); c_1 = 40 x 10^{-6} M; c_2 = 1.5 x 10^{-6} M (l); c_1 = 20 x 10^{-6} M; c_2 = 3.75 x 10^{-6} M (m); c_1 = 40 x 10^{-6} M; c_2 = 7.5 x 10^{-6} M (n); c_1 = 80 x 10^{-6} M; c_2 = 15 x 10^{-6} M (o); c_1 = 40 x 10^{-6} M; c_2 = 2 x 10^{-6} M (p); c_1 = 20 x 10^{-6} M; c_2 = 5 x 10^{-6} M (q); c_1 = 40 x 10^{-6} M; c_2 = 10 x 10^{-6} M (r); c_1 = 80 x 10^{-6} M; c_2 = 20 x 10^{-6} M (s); c_1 = 40 x 10^{-6} M; c_2 = 40 x 10^{-6} M (t); c_1 = 25 x 10^{-6} M; c_2 = 5 x 10^{-6} M (u); c_1 = 30 x 10^{-6} M; c_2 = 5 x 10^{-6} M (v); c_1 = 35 x 10^{-6} M; c_2 = 5 x 10^{-6} M (w); c_1 = 40 x 10^{-6} M; c_2 = 5 x 10^{-6} M (x).

7 days [**1**]:[**2**] 10^{-6} M

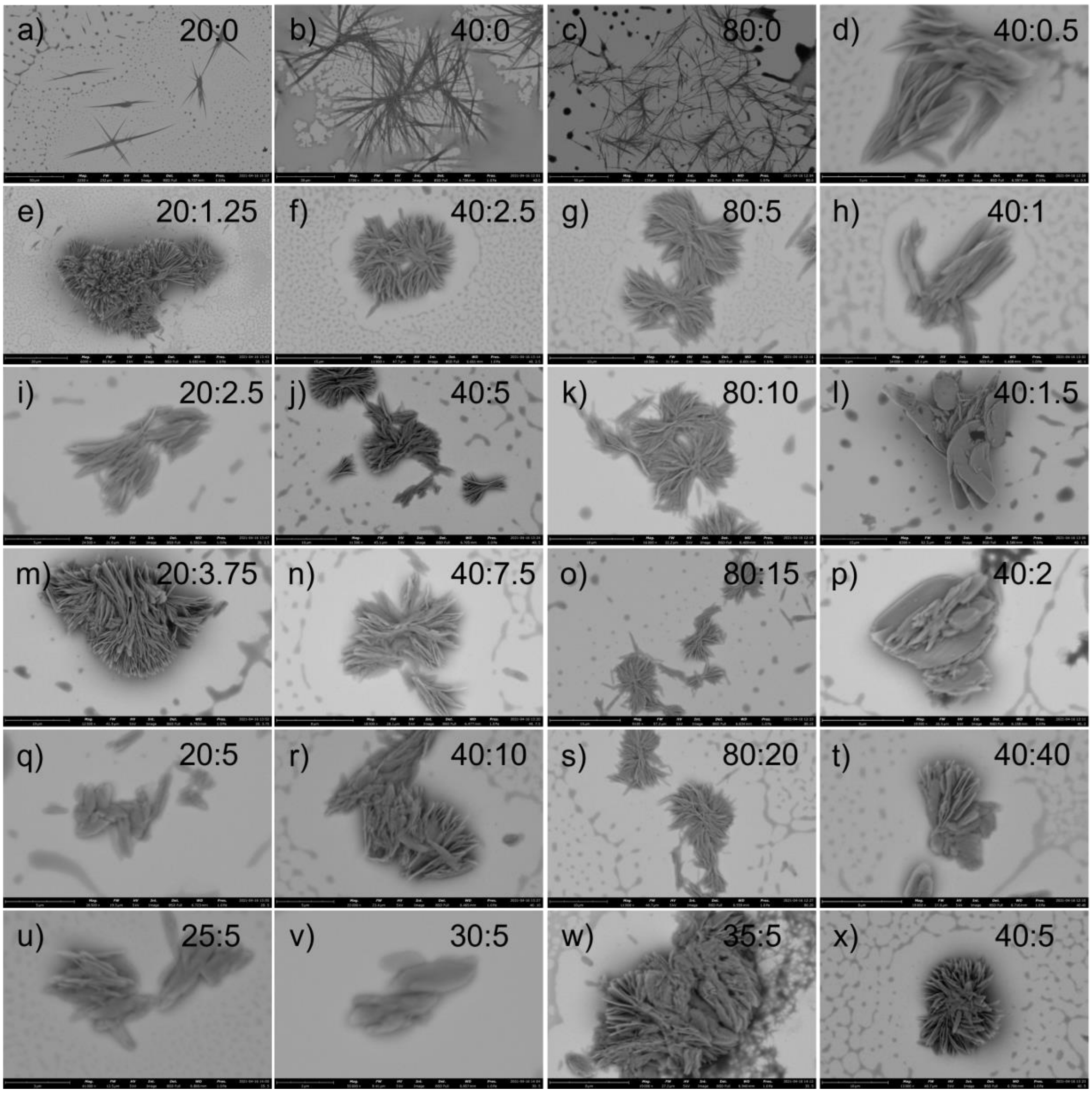

Supplementary Figure 33: a-x) SEM micrographs of Agg**1** seven days after applying the kinetic self-assembly protocol described in the extended information; the specific ratio between **1** and **2** used to investigate the morphology upon crowding were: c_1 = 20 x 10^{-6} M; c_2 = 0 x 10^{-6} M (a); c_1 = 40 x 10^{-6} M; c_2 = 0 x 10^{-6} M (b); c_1 = 80 x 10^{-6} M; c_2 = 0 x 10^{-6} M (c); c_1 = 40 x 10^{-6} M; c_2 = 0.5 x 10^{-6} M (d); c_1 = 20 x 10^{-6} M; c_2 = 1.25 x 10^{-6} M (e); c_1 = 40 x 10^{-6} M; c_2 = 2.5 x 10^{-6} M (f); c_1 = 80 x 10^{-6} M; c_2 = 5 x 10^{-6} M (g); c_1 = 40 x 10^{-6} M; c_2 = 1 x 10^{-6} M (h); c_1 = 20 x 10^{-6} M; c_2 = 2.5 x 10^{-6} M (i); c_1 = 40 x 10^{-6} M; c_2 = 5 x 10^{-6} M (j); c_1 = 80 x 10^{-6} M; c_2 = 10 x 10^{-6} M (k); c_1 = 40 x 10^{-6} M; c_2 = 1.5 x 10^{-6} M (l); c_1 = 20 x 10^{-6} M; c_2 = 3.75 x 10^{-6} M (m); c_1 = 40 x 10^{-6} M; c_2 = 7.5 x 10^{-6} M (n); c_1 = 80 x 10^{-6} M; c_2 = 15 x 10^{-6} M (o); c_1 = 40 x 10^{-6} M; c_2 = 2 x 10^{-6} M (p); c_1 = 20 x 10^{-6} M; c_2 = 5 x 10^{-6} M (q); c_1 = 40 x 10^{-6} M; c_2 = 10 x 10^{-6} M (r); c_1 = 80 x 10^{-6} M; c_2 = 20 x 10^{-6} M (s); c_1 = 40 x 10^{-6} M; c_2 = 40 x 10^{-6} M (t); c_1 = 25 x 10^{-6} M; c_2 = 5 x 10^{-6} M (u); c_1 = 30 x 10^{-6} M; c_2 = 5 x 10^{-6} M (v); c_1 = 35 x 10^{-6} M; c_2 = 5 x 10^{-6} M (w); c_1 = 40 x 10^{-6} M; c_2 = 5 x 10^{-6} M (x).

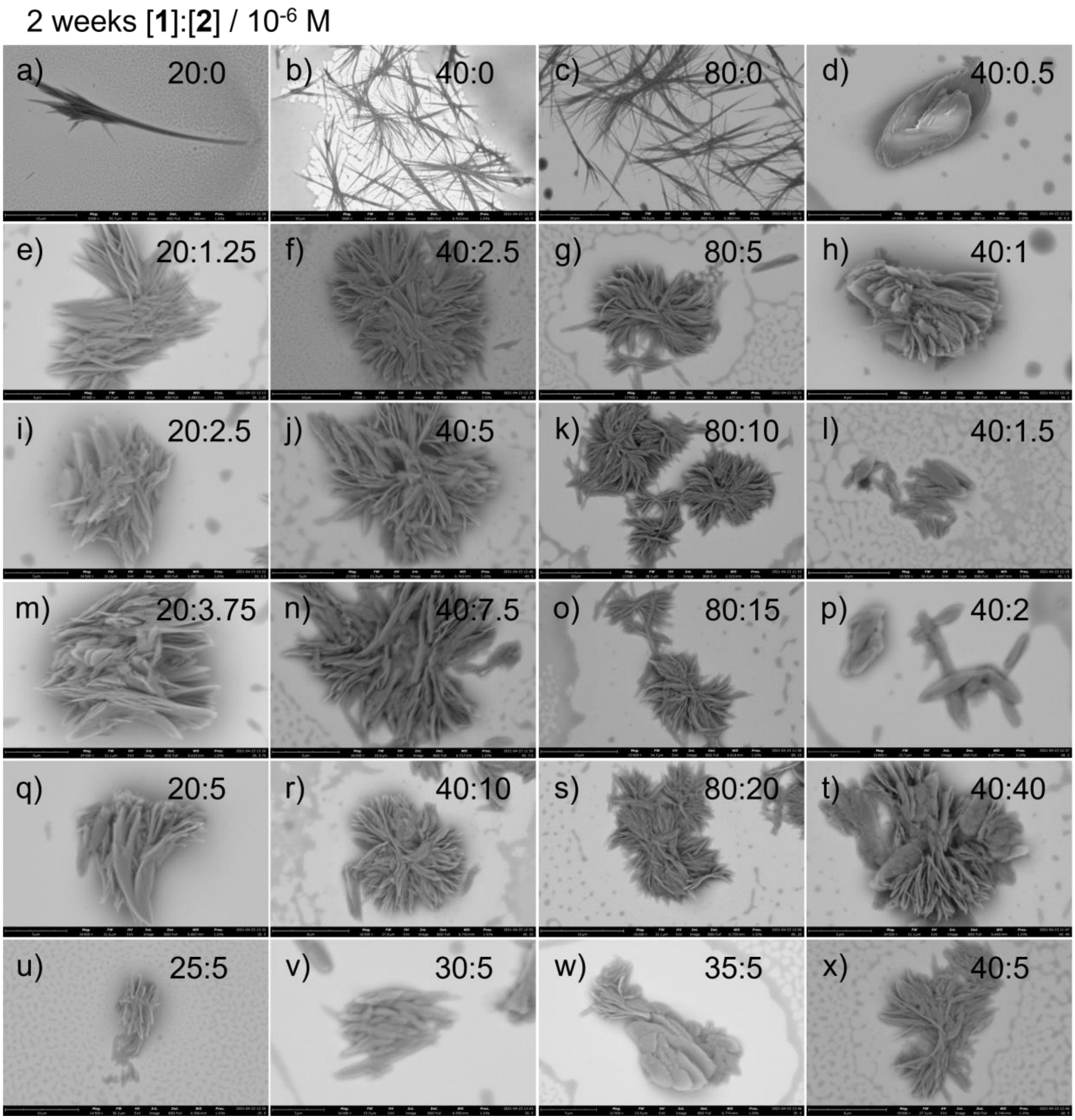

Supplementary Figure 34: a-x) SEM micrographs of Agg**1** two weeks after applying the kinetic self-assembly protocol described in the extended information; the specific ratio between **1** and **2** used to investigate the morphology upon crowding were: c_1 = 20 x 10^{-6} M; c_2 = 0 x 10^{-6} M (a); c_1 = 40 x 10^{-6} M; c_2 = 0 x 10^{-6} M (b); c_1 = 80 x 10^{-6} M; c_2 = 0 x 10^{-6} M (c); c_1 = 40 x 10^{-6} M; c_2 = 0.5 x 10^{-6} M (d); c_1 = 20 x 10^{-6} M; c_2 = 1.25 x 10^{-6} M (e); c_1 = 40 x 10^{-6} M; c_2 = 2.5 x 10^{-6} M (f); c_1 = 80 x 10^{-6} M; c_2 = 5 x 10^{-6} M (g); c_1 = 40 x 10^{-6} M; c_2 = 1 x 10^{-6} M (h); c_1 = 20 x 10^{-6} M; c_2 = 2.5 x 10^{-6} M (i); c_1 = 40 x 10^{-6} M; c_2 = 5 x 10^{-6} M (j); c_1 = 80 x 10^{-6} M; c_2 = 10 x 10^{-6} M (k); c_1 = 40 x 10^{-6} M; c_2 = 1.5 x 10^{-6} M (l); c_1 = 20 x 10^{-6} M; c_2 = 3.75 x 10^{-6} M (m); c_1 = 40 x 10^{-6} M; c_2 = 7.5 x 10^{-6} M (n); c_1 = 80 x 10^{-6} M; c_2 = 15 x 10^{-6} M (o); c_1 = 40 x 10^{-6} M; c_2 = 2 x 10^{-6} M (p); c_1 = 20 x 10^{-6} M; c_2 = 5 x 10^{-6} M (q); c_1 = 40 x 10^{-6} M; c_2 = 10 x 10^{-6} M (r); c_1 = 80 x 10^{-6} M; c_2 = 20 x 10^{-6} M (s); c_1 = 40 x 10^{-6} M; c_2 = 40 x 10^{-6} M (t); c_1 = 25 x 10^{-6} M; c_2 = 5 x 10^{-6} M (u); c_1 = 30 x 10^{-6} M; c_2 = 5 x 10^{-6} M (v); c_1 = 35 x 10^{-6} M; c_2 = 5 x 10^{-6} M (w); c_1 = 40 x 10^{-6} M; c_2 = 5 x 10^{-6} M (x).

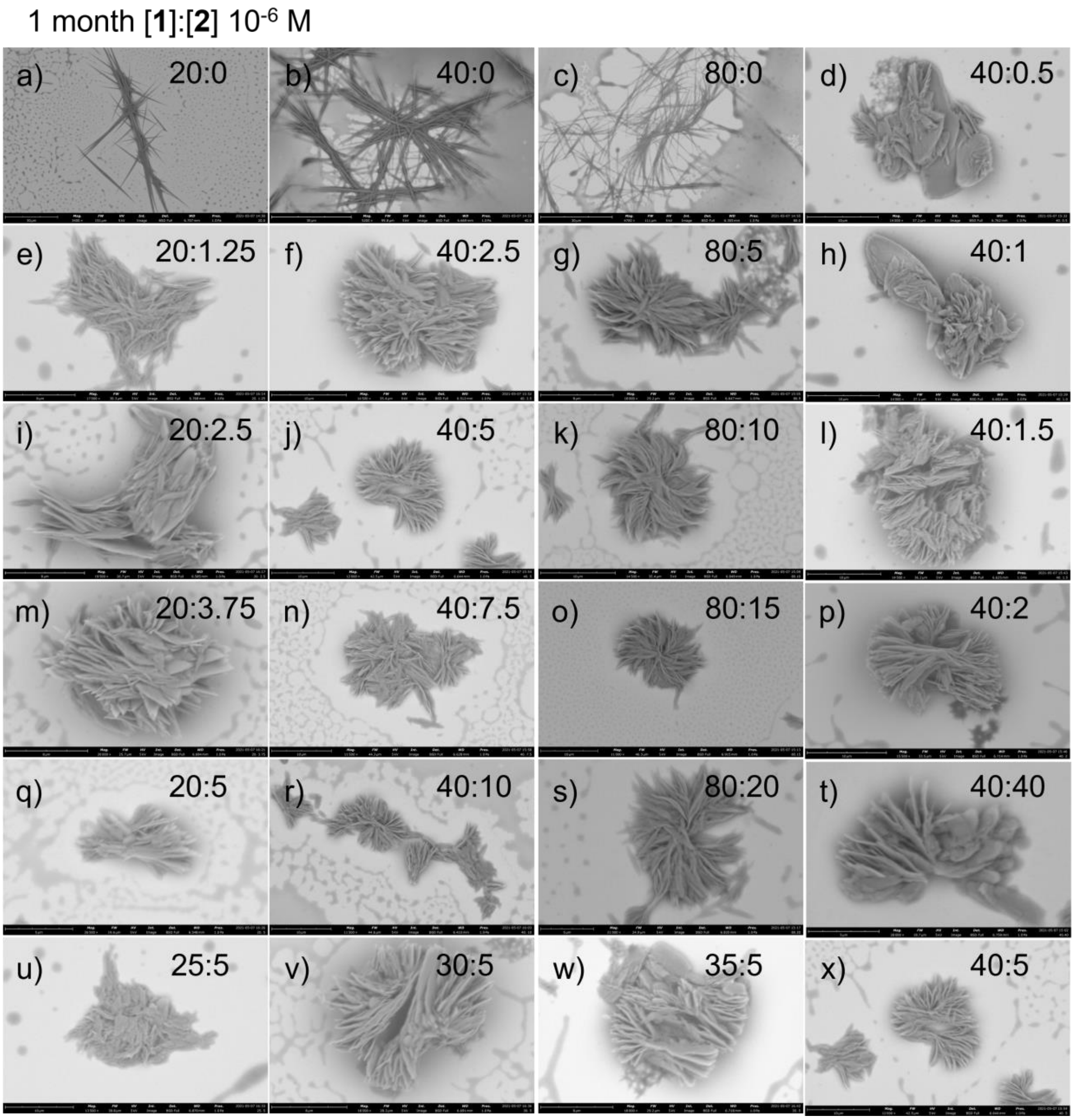

Supplementary Figure 35: a-x) SEM micrographs of Agg**1** one month after applying the kinetic self-assembly protocol described in the extended information; the specific ratio between **1** and **2** used to investigate the morphology upon crowding were: c_1 = 20 x 10^{-6} M; c_2 = 0 x 10^{-6} M (a); c_1 = 40 x 10^{-6} M; c_2 = 0 x 10^{-6} M (b); c_1 = 80 x 10^{-6} M; c_2 = 0 x 10^{-6} M (c); c_1 = 40 x 10^{-6} M; c_2 = 0.5 x 10^{-6} M (d); c_1 = 20 x 10^{-6} M; c_2 = 1.25 x 10^{-6} M (e); c_1 = 40 x 10^{-6} M; c_2 = 2.5 x 10^{-6} M (f); c_1 = 80 x 10^{-6} M; c_2 = 5 x 10^{-6} M (g); c_1 = 40 x 10^{-6} M; c_2 = 1 x 10^{-6} M (h); c_1 = 20 x 10^{-6} M; c_2 = 2.5 x 10^{-6} M (i); c_1 = 40 x 10^{-6} M; c_2 = 5 x 10^{-6} M (j); c_1 = 80 x 10^{-6} M; c_2 = 10 x 10^{-6} M (k); c_1 = 40 x 10^{-6} M; c_2 = 1.5 x 10^{-6} M (l); c_1 = 20 x 10^{-6} M; c_2 = 3.75 x 10^{-6} M (m); c_1 = 40 x 10^{-6} M; c_2 = 7.5 x 10^{-6} M (n); c_1 = 80 x 10^{-6} M; c_2 = 15 x 10^{-6} M (o); c_1 = 40 x 10^{-6} M; c_2 = 2 x 10^{-6} M (p); c_1 = 20 x 10^{-6} M; c_2 = 5 x 10^{-6} M (q); c_1 = 40 x 10^{-6} M; c_2 = 10 x 10^{-6} M (r); c_1 = 80 x 10^{-6} M; c_2 = 20 x 10^{-6} M (s); c_1 = 40 x 10^{-6} M; c_2 = 40 x 10^{-6} M (t); c_1 = 25 x 10^{-6} M; c_2 = 5 x 10^{-6} M (u); c_1 = 30 x 10^{-6} M; c_2 = 5 x 10^{-6} M (v); c_1 = 35 x 10^{-6} M; c_2 = 5 x 10^{-6} M (w); c_1 = 40 x 10^{-6} M; c_2 = 5 x 10^{-6} M (x).

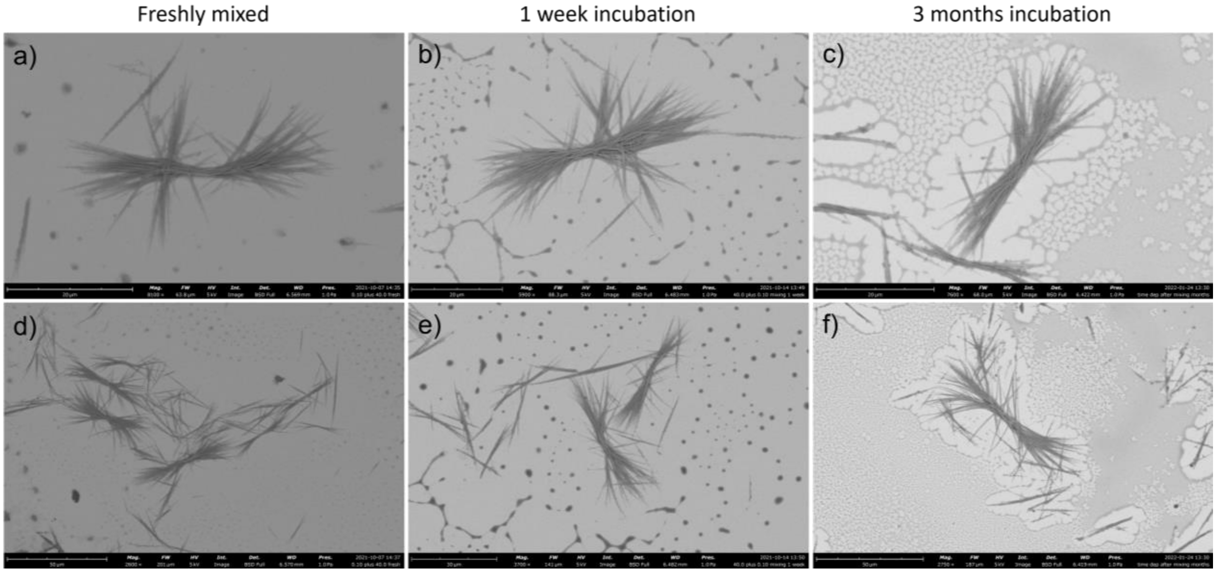

Supplementary Figure 36: a-f) Time-dependent SEM micrographs of Agg**1U** after mixing with Agg**2** under ambient conditions highlighting the kinetic stability of Agg**1U** under these conditions. Images recorded immediately after mixing (a,d), after one week (b,e) and after three months (c,f).

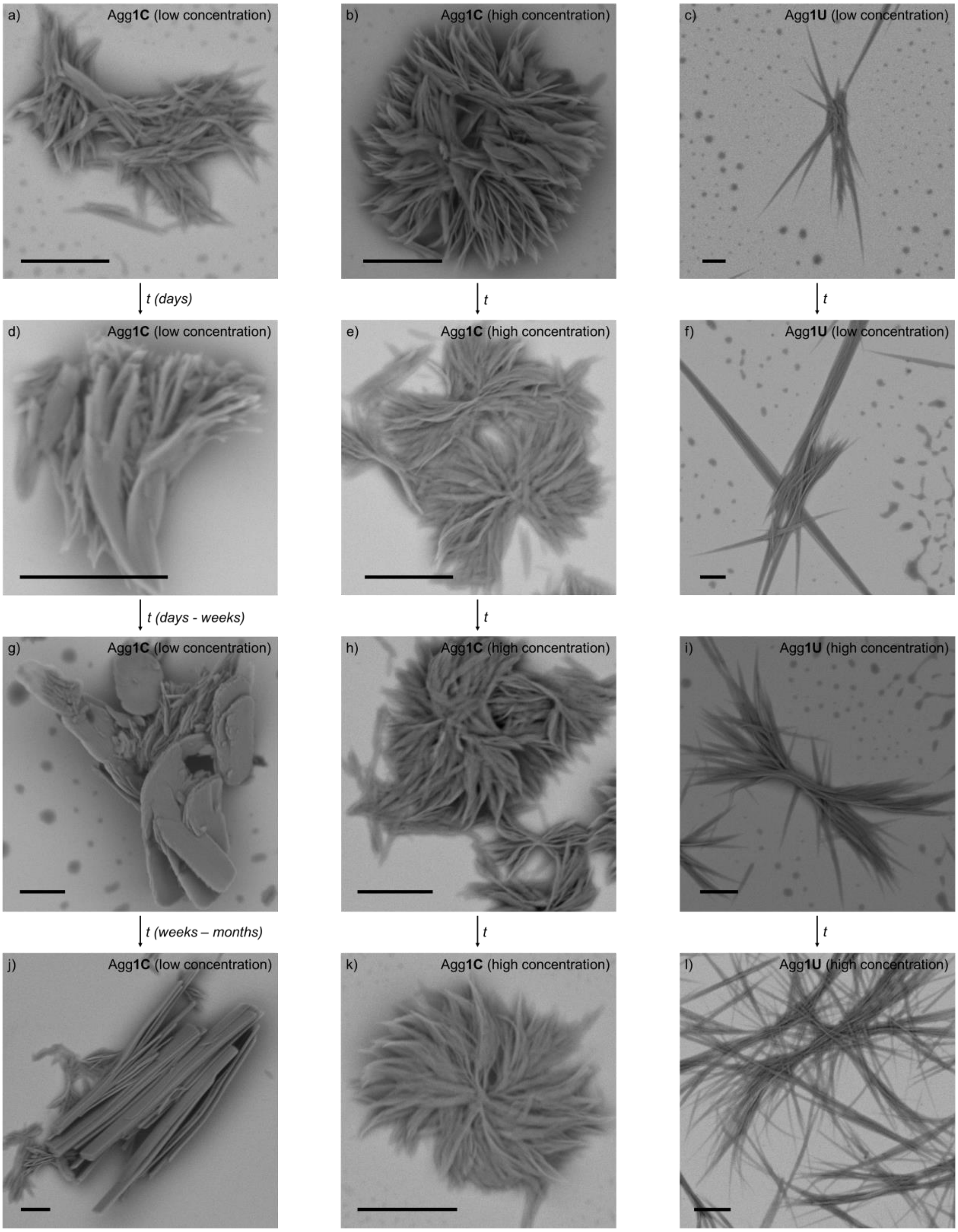

Supplementary Figure 37: a,d,g,j) Representative SEM micrographs depicting the time-dependent rearrangement of the flower-like structures of Agg**1C** at low concentration into ribbon-like structures with a pronounced hierarchical order ($c_1 \leq 40 \times 10^{-6}$ M). b,e,h,k) Representative SEM micrographs depicting the inability of Agg**1C** to rearrange into higher order structures at high concentrations. Time-dependent rearrangement of the flower-like structures of Agg**1C** at low concentration into ribbon-like structures with a pronounced hierarchical order ($c_1 = 80 \times 10^{-6}$ M). c,f,i,l) Representative SEM micrographs depicting the inability of Agg**1U** to rearrange into higher ordered structures irrespective of the concentration ($c_1 = 20 \times 10^{-6}$ M (c,f); 80×10^{-6} M (i,l)). All scale bars correspond to 5 µm. For additional SEM micrographs, please see Supplementary Figure 32-35.

Seed-induced supramolecular polymerization experiments in crowded and uncrowded environments

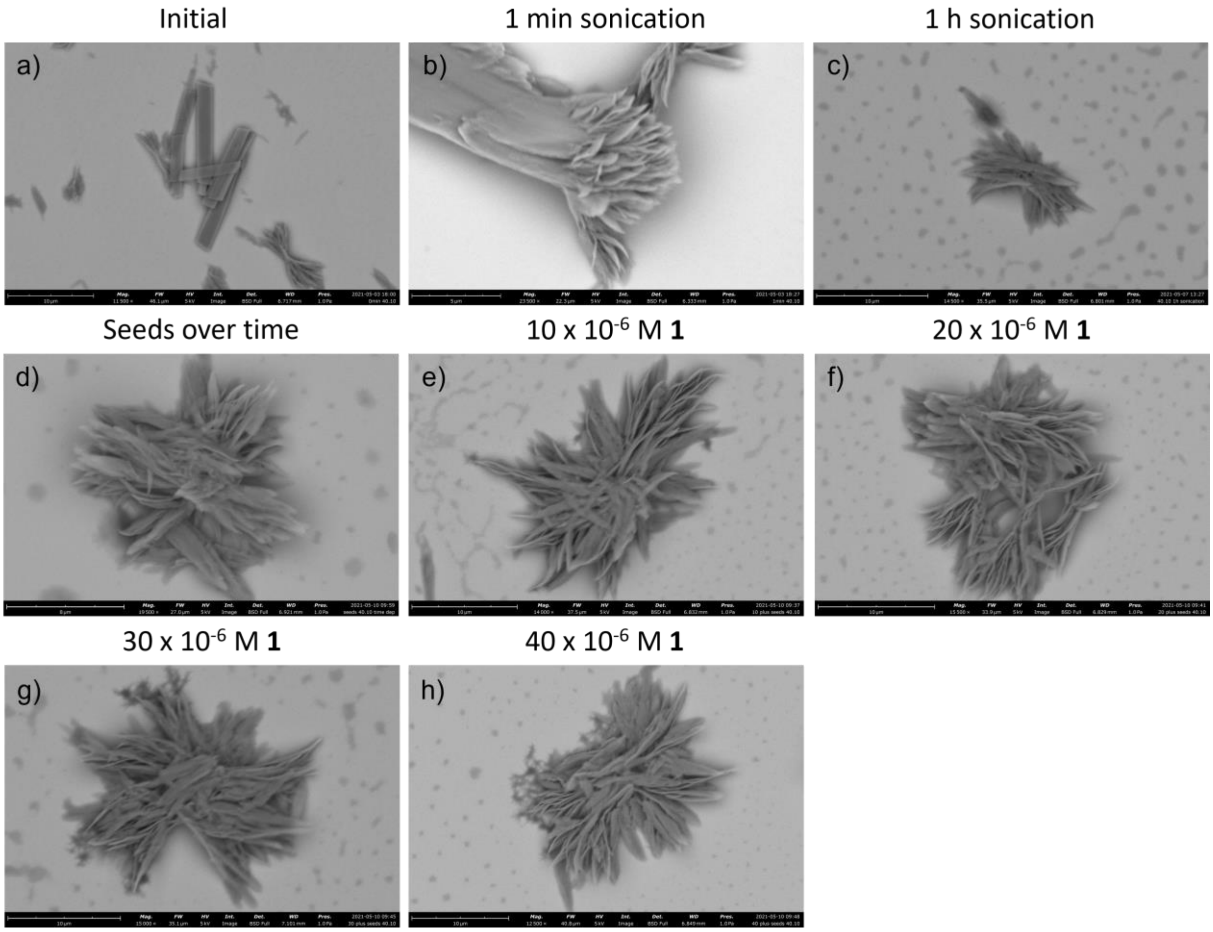

Supplementary Figure 38: a-c) SEM micrographs of Agg**1C** (c_1 = 40 x 10^{-6} M, c_2 = 10 x 10^{-6} M) after 2 months of incubation at room temperature (a) and subsequent sonication(b,c). d-h) SEM micrographs of the seeds without (d) and with addition of aliquots of **1** following three days of incubation (c_1 = 10 x 10^{-6} M (e); c_1 = 20 x 10^{-6} M (f); c_1 = 30 x 10^{-6} M (g); c_1 = 40 x 10^{-6} M (h)), revealing no apparent changes in the morphology as observed for the pure seeds after the same time period.

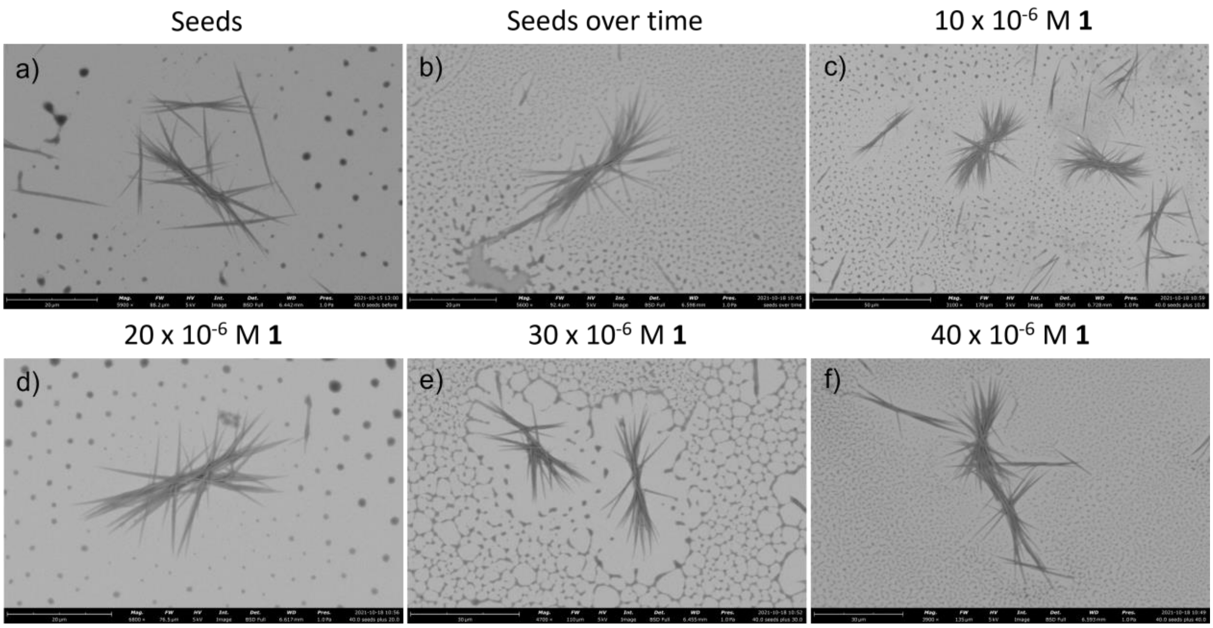

Supplementary Figure 39: a+b) SEM micrographs of Agg**1U** (c_1 = 40 x 10^{-6} M) used as seeds (a) for seed induced self-assembly, with the seeds after three days depicted in (b). c-f) SEM micrographs of the seeds after addition of aliquots of **1** (c_1 = 10 x 10^{-6} M (c); c_1 = 20 x 10^{-6} M (d); c_1 = 30 x 10^{-6} M (e); c_1 = 40 x 10^{-6} M (f)) following three days of incubation, revealing no apparent changes in the morphology as observed for the pure seeds after the same time period.

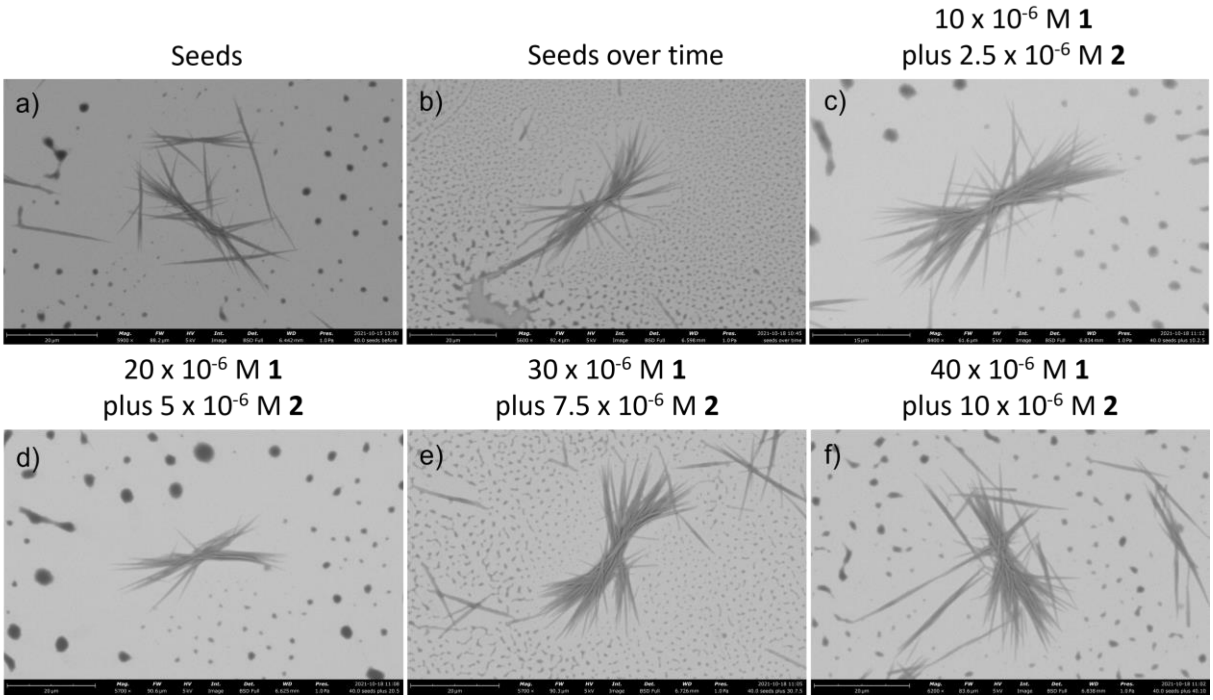

Supplementary Figure 40: a+b) SEM micrographs of Agg**1U** (c_1 = 40 x 10^{-6} M) used as seeds (a) for seed induced self-assembly, with the seeds after three days depicted in (b). c-f) SEM micrographs of the seeds after addition of aliquots of **1** in a crowded environment (c_1 = 10 x 10^{-6} M, c_2 = 2.5 x 10^{-6} M (c); c_1 = 20 x 10^{-6} M, c_2 = 5 x 10^{-6} M (d); c_1 = 30 x 10^{-6} M, c_2 = 7.5 x 10^{-6} M (e); c_1 = 40 x 10^{-6} M, c_2 = 10 x 10^{-6} M (f)) following three days of incubation, revealing no apparent changes in the morphology as observed for the pure seeds after the same time period.

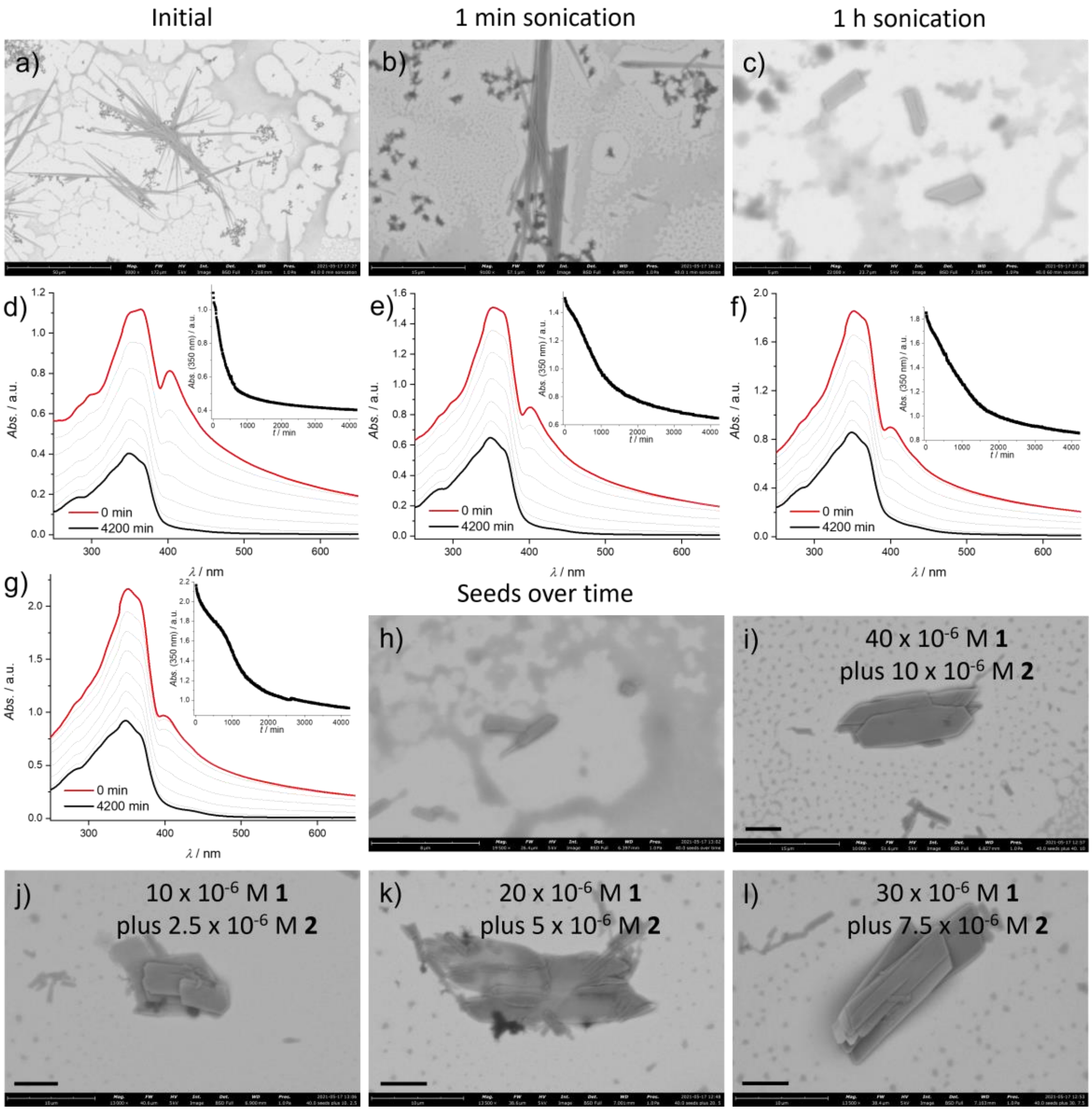

Supplementary Figure 41: a-c,h) SEM micrographs highlighting the seed formation (a-c, c_1 = 40 x 10^{-6} M) and the seed stability over time (h). d-g) Time-dependent UV/Vis spectra of mixtures of **1** and **2** after addition of seeds of Agg**1T** with the time-dependent absorbance changes at the absorption maximum (λ = 350 nm) depicted as insets using the seeding protocol described in the method section; c_1 = 10 x 10^{-6} M, c_2 = 2.5 x 10^{-6} M (d); c_1 = 20 x 10^{-6} M, c_2 = 5 x 10^{-6} M (e); c_1 = 30 x 10^{-6} M, c_2 = 7.5 x 10^{-6} M (f); c_1 = 40 x 10^{-6} M, c_2 = 10 x 10^{-6} M (g). i-l) Agg**1Ts** after addition of aliquots of **1** in a crowded environment.

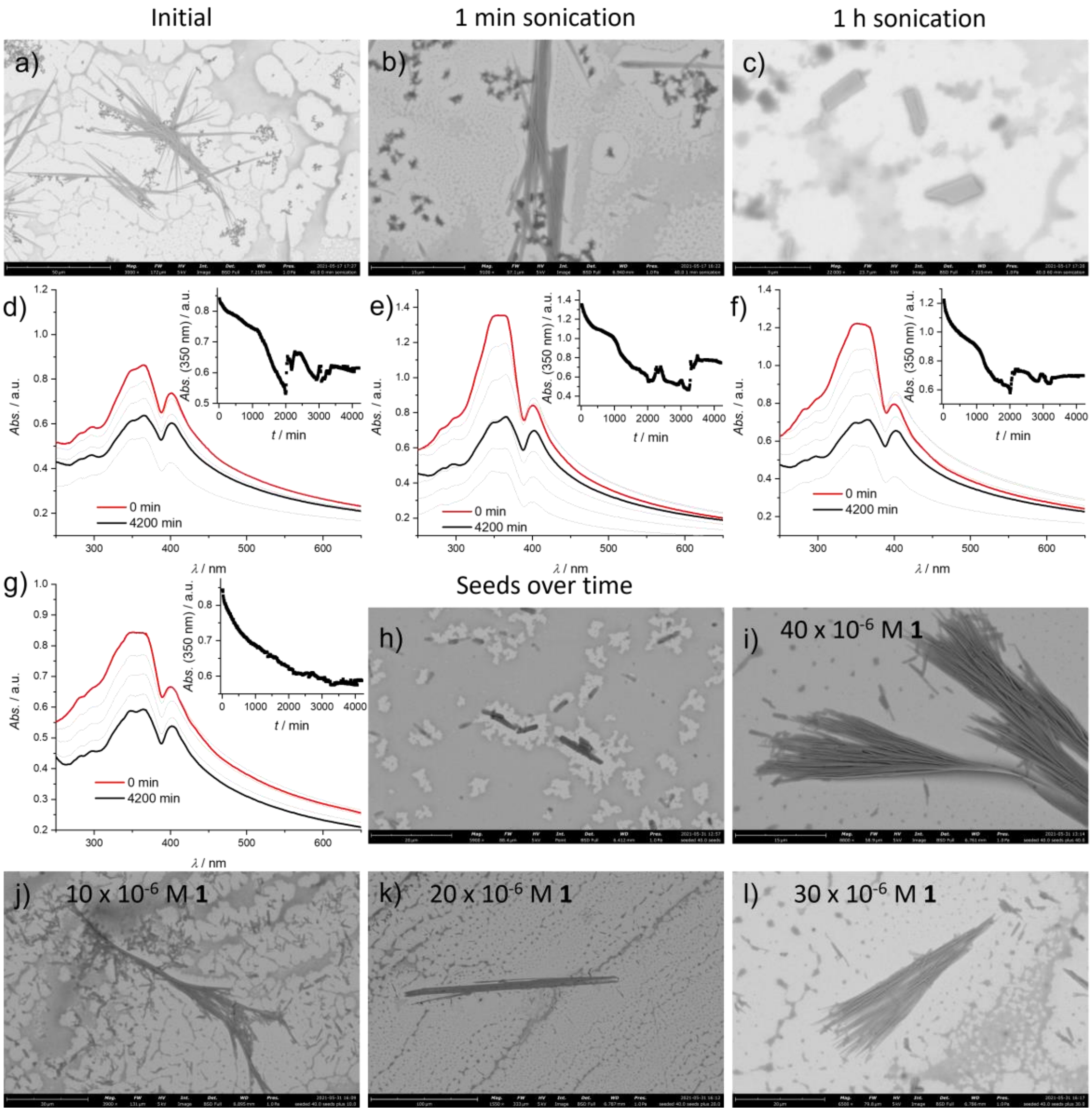

Supplementary Figure 42: a-c,h) SEM micrographs highlighting the seed formation (a-c, c_1 = 40 x 10^{-6} M) and the seed stability over time (h). d-g) Time-dependent UV/Vis spectra of mixtures of **1** and **2** after addition of seeds of Agg**1T** with the time-dependent absorbance changes at the absorption maximum (λ = 350 nm) depicted as insets using the seeding protocol described in the method section; c_1 = 10 x 10^{-6} M (d); c_1 = 20 x 10^{-6} M (e); c_1 = 30 x 10^{-6} M (f); c_1 = 40 x 10^{-6} M (g) i-l) Agg**1Us** after addition of aliquots of **1** in an uncrowded environment.

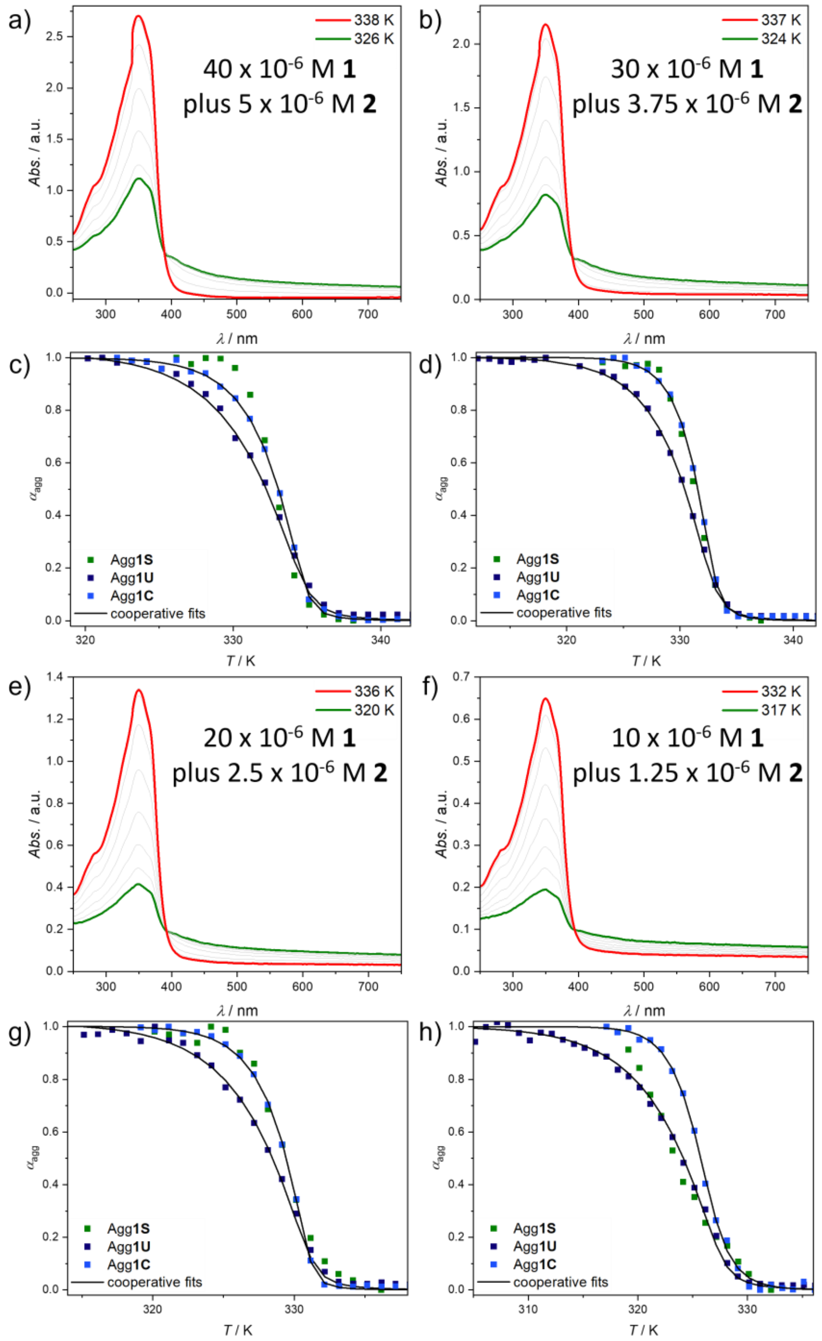

Supplementary Figure 43: (a,b,e,f) Temperature-dependent UV/Vis spectra of Agg**1Ts** following the heating protocol described in the method section, using a range of different concentrations with a visual comparison between the experimental heating curves for Agg**1Ts**, Agg**1U** and Agg**1C**, using identical concentrations of **1** as presented in the corresponding UV/Vis experiment depicted above (c,d,g,h).

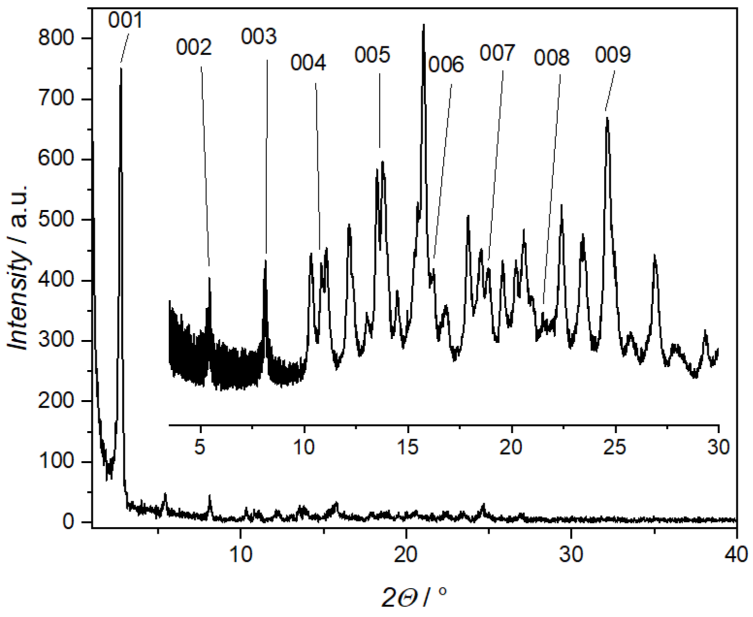

Supplementary Figure 44: XRD patterns of Agg**1Ts** between 2 and 40° with an inset depicting the range between 3 and 30°.

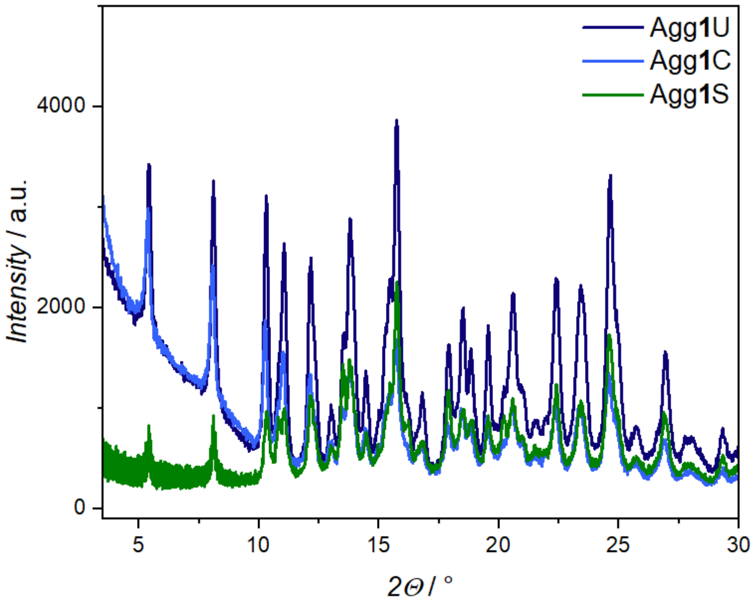

Supplementary Figure 45: XRD patterns of Agg**1U**, Agg**1C** and Agg**1Ts** between 3 and 30°.

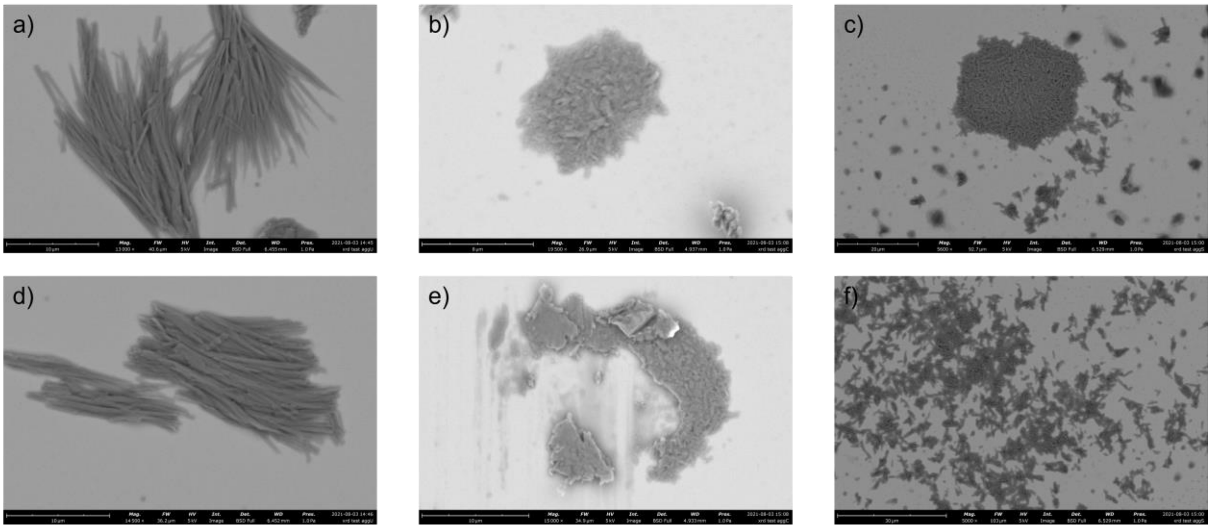

Supplementary Figure 46: a-f) SEM micrographs of the different aggregates after the XRD measurements highlighting the structure stability to the employed sample preparation protocol. Agg**1U** appears as fractured bundles of fibres (a,d), Agg**1C** shows the typical 2D sheet-like structure previously observed in the AFM (b,e) and Agg**1Ts** could be observed as short ribbons bundled together (c,f).

Self-assembly of 1 in the presence of distinct crowding agents

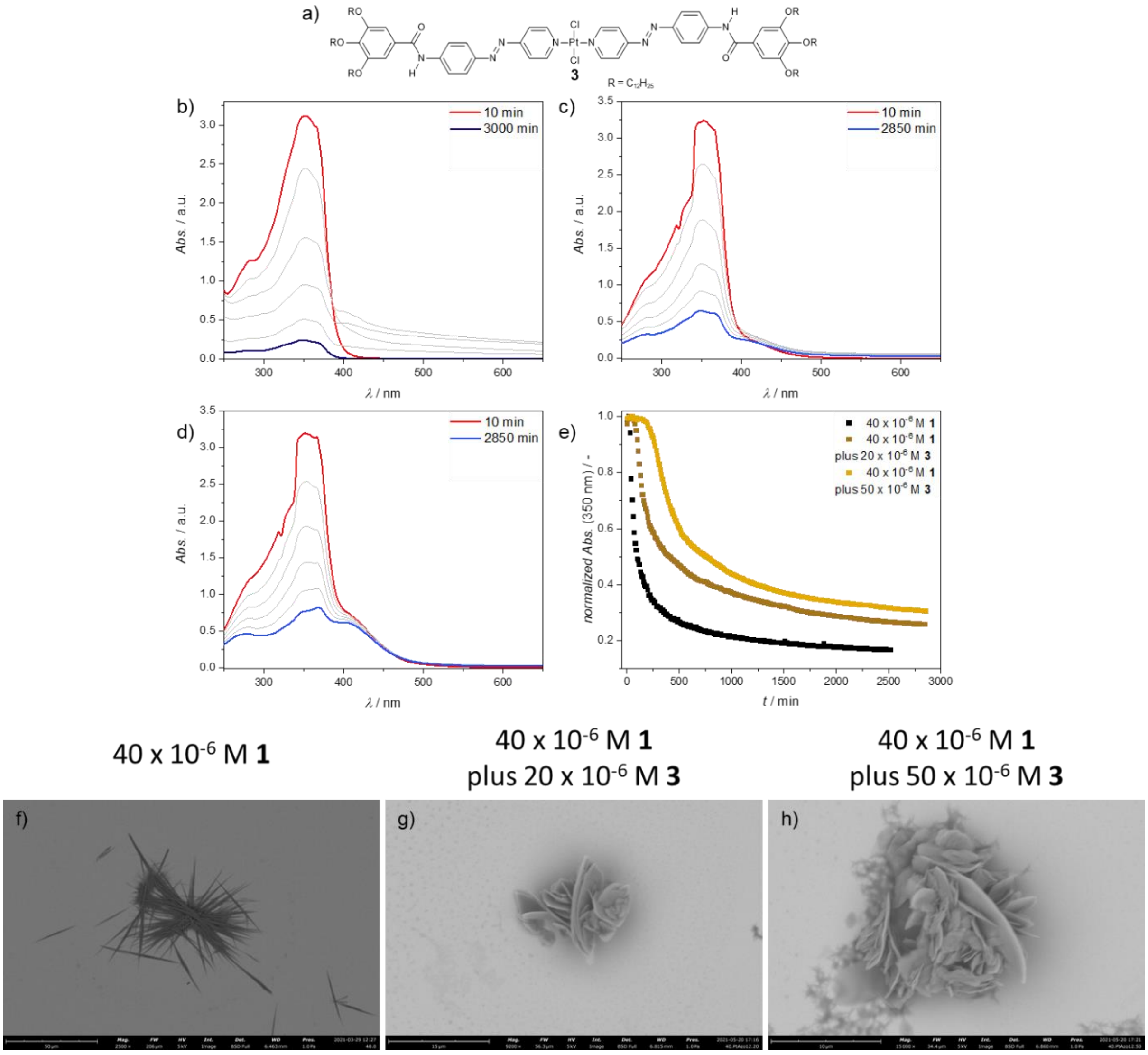

Supplementary Figure 47: a) Chemical structure of **3**. b-e) Time-dependent UV/Vis spectra of mixtures of **1** and **3** using a concentration of 40 x 10^{-6} M of **1** and varying amounts of **3** (0 (b), 20 (c) and 50 x 10^{-6} M (d)) with the time-dependent absorbance changes at the absorption maximum of **1** (λ = 350 nm) depicted in (e). f-h) SEM micrographs of Agg**1** obtained after the time-dependent UV/Vis studies.

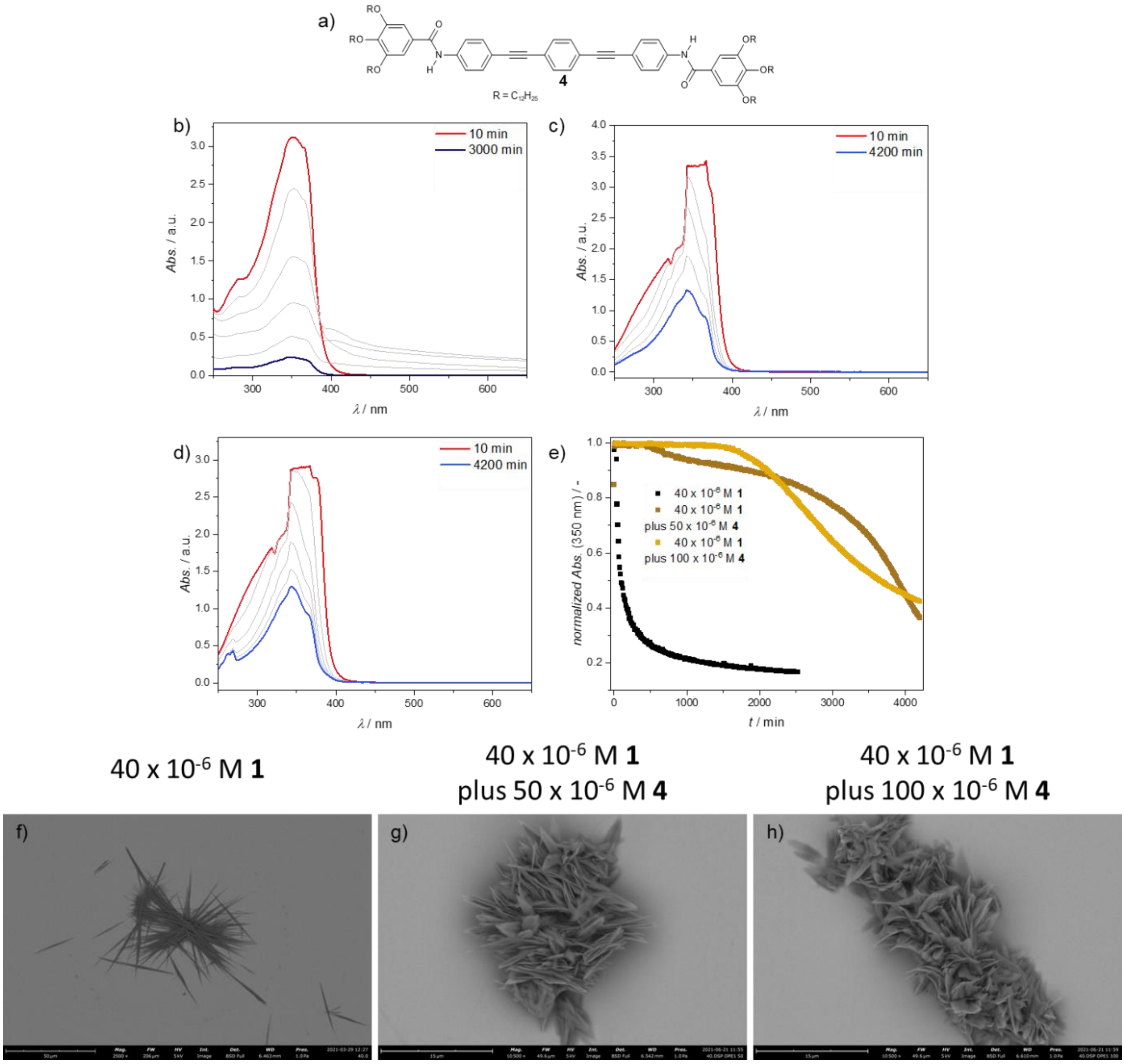

Supplementary Figure 48: a) Chemical structure of **4**. b-e) Time-dependent UV/Vis spectra of mixtures of **1** and **4** using a concentration of 40 x 10^{-6} M of **1** and varying amounts of **4** (0 (b), 50 (c) and 100 x 10^{-6} M (d)) with the time-dependent absorbance changes at the absorption maximum of **1** (λ = 350 nm) depicted in (e). f-h) SEM micrographs of Agg**1** obtained after the time-dependent UV/Vis studies.

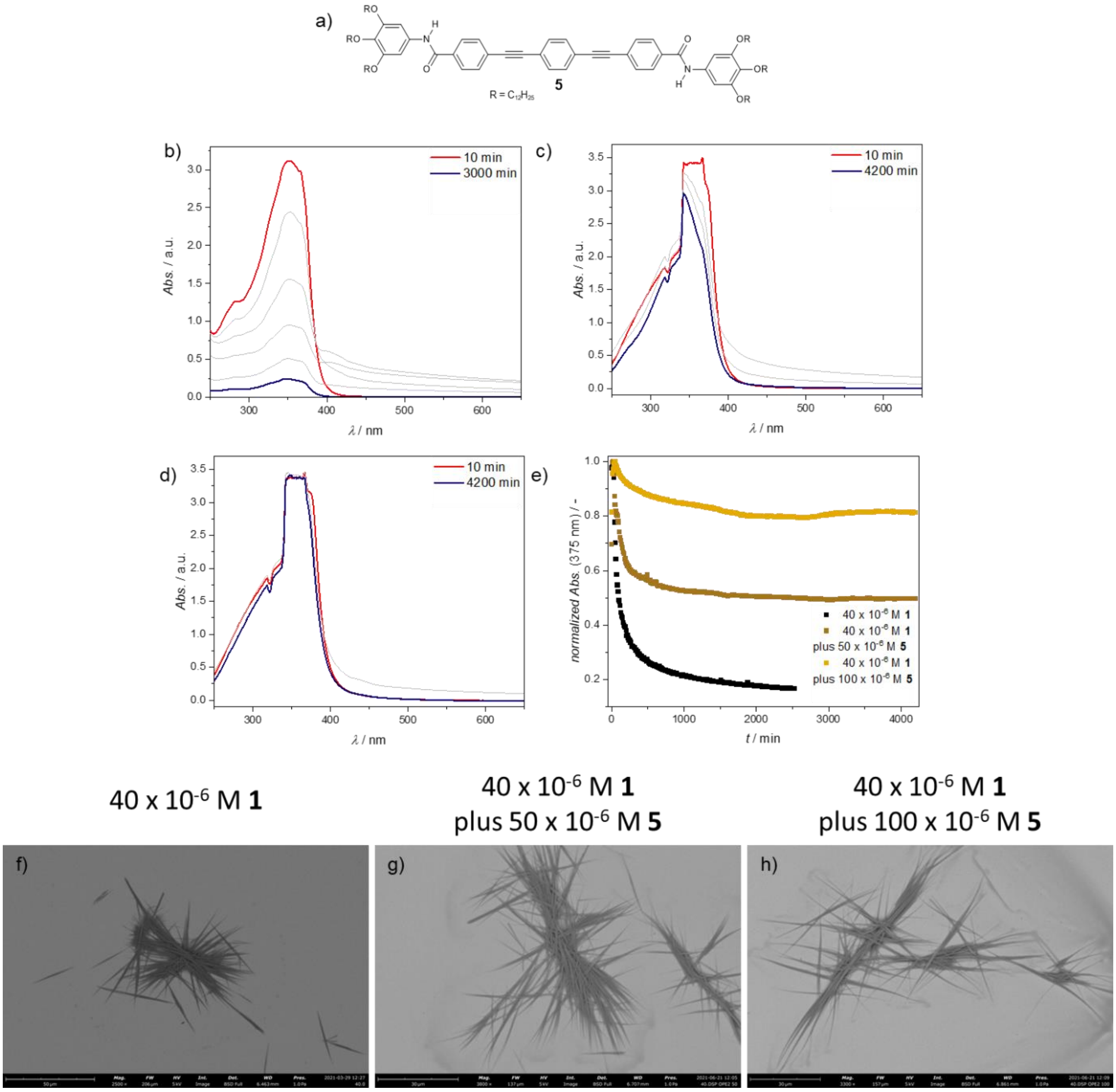

Supplementary Figure 49: a) Chemical structure of **5**. b-e) Time-dependent UV/Vis spectra of mixtures of **1** and **5** using a concentration of 40 x 10^{-6} M of **1** and varying amounts of **5** (0 (b), 50 (c) and 100 x 10^{-6} M (d)) with the time-dependent absorbance changes at λ = 375 nm depicted in (e). f-h) SEM micrographs of Agg**1** obtained after the time-dependent UV/Vis studies.

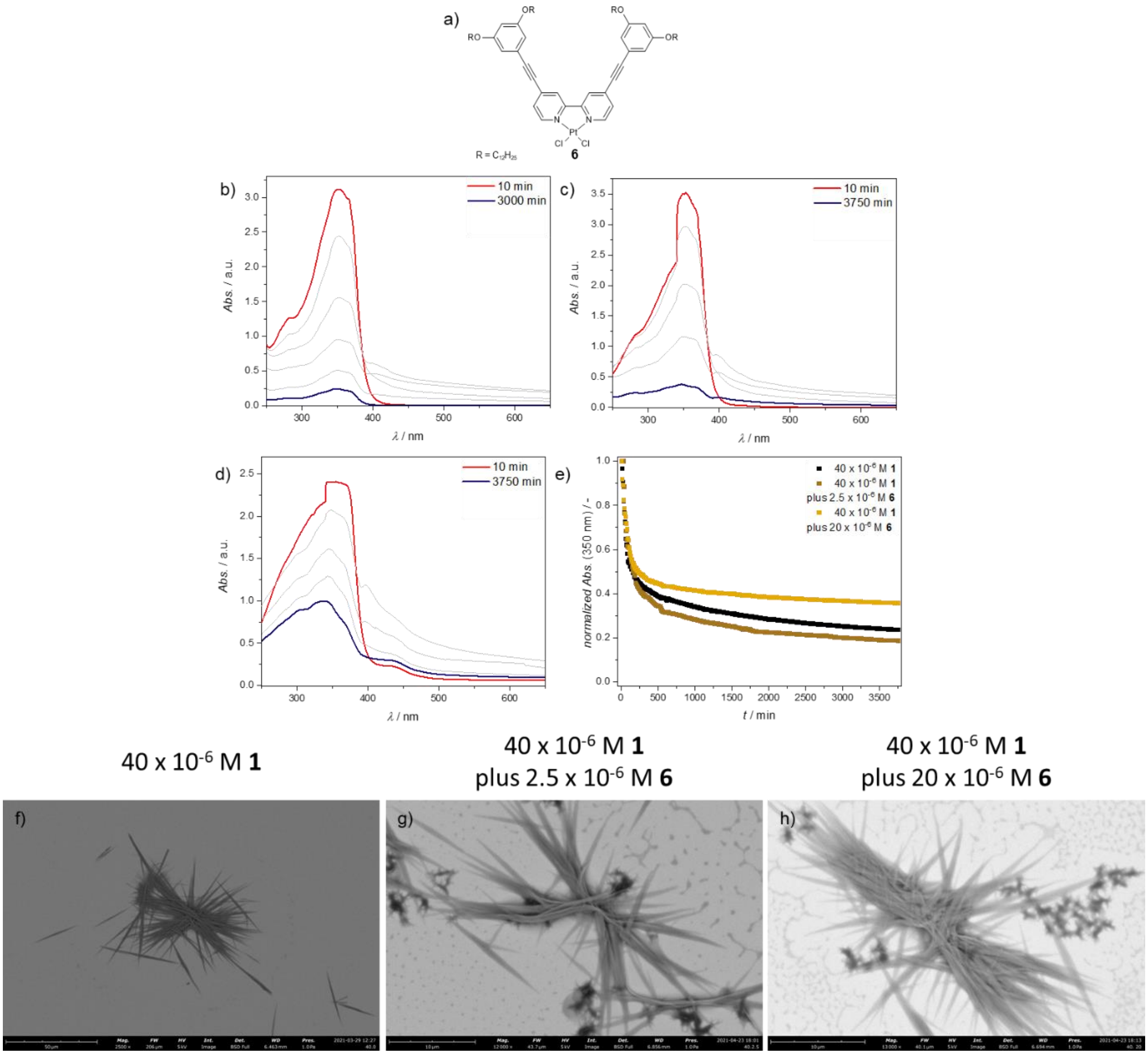

Supplementary Figure 50: a) Chemical structure of **6**. b-e) Time-dependent UV/Vis spectra of mixtures of **1** and **6** using a concentration of 40 x 10^{-6} M of **1** and varying amounts of **6** (0 (b), 2.5 (c) and 20 x 10^{-6} M (d)) with the time-dependent absorbance changes at the absorption maximum of **1** (λ = 350 nm) depicted in (e). f-h) SEM micrographs of Agg**1** obtained after the time-dependent UV/Vis studies.

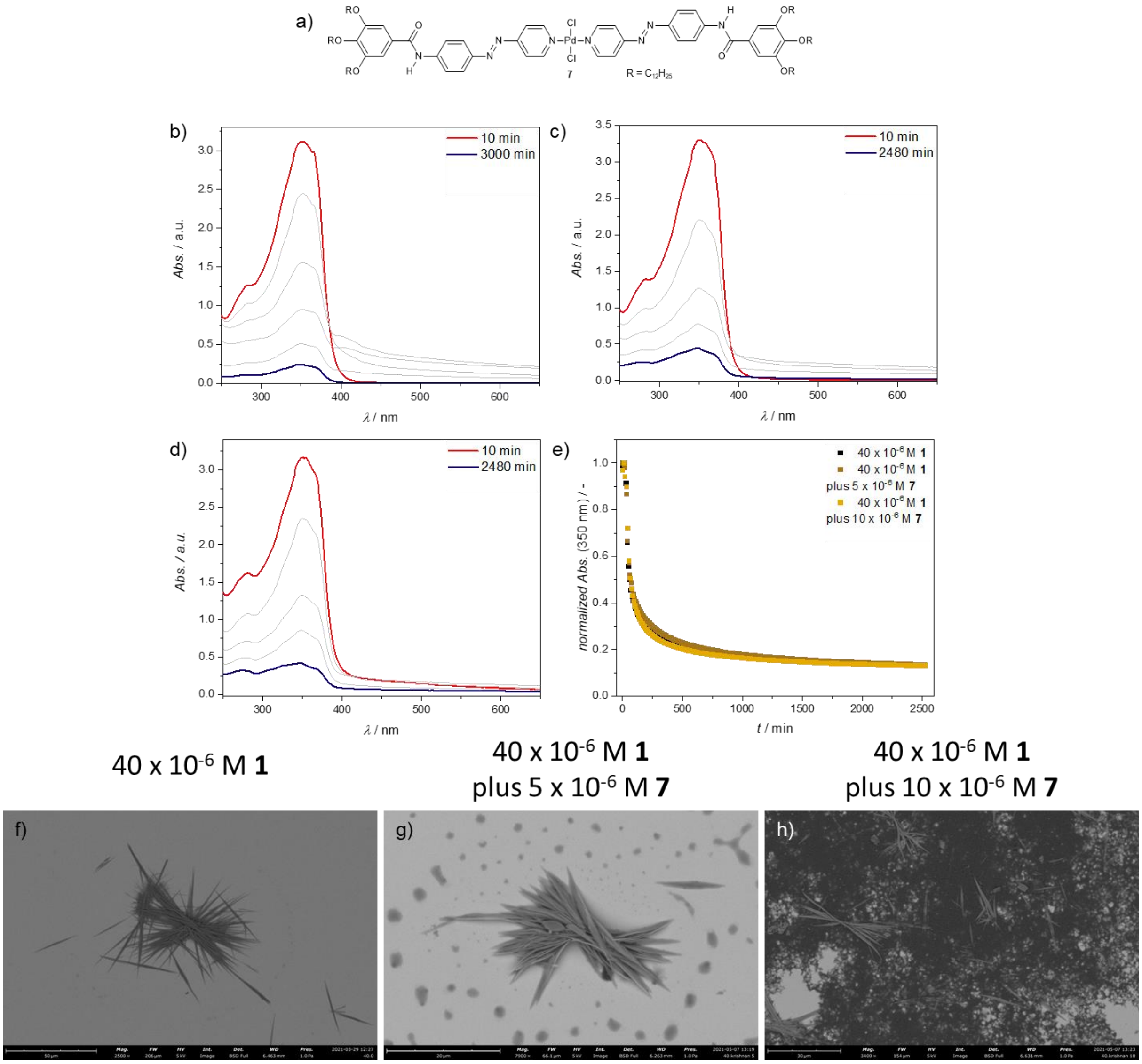

Supplementary Figure 51: a) Chemical structure of **7**. b-e) Time-dependent UV/Vis spectra of mixtures of **1** and **7** using a concentration of 40 x 10^{-6} M of **1** and varying amounts of **7** (0 (b), 5 (c) and 10 x 10^{-6} M (d)) with the time-dependent absorbance changes at the absorption maximum of **1** (λ = 350 nm) depicted in (e). f-h) SEM micrographs of Agg**1** obtained after the time-dependent UV/Vis studies.

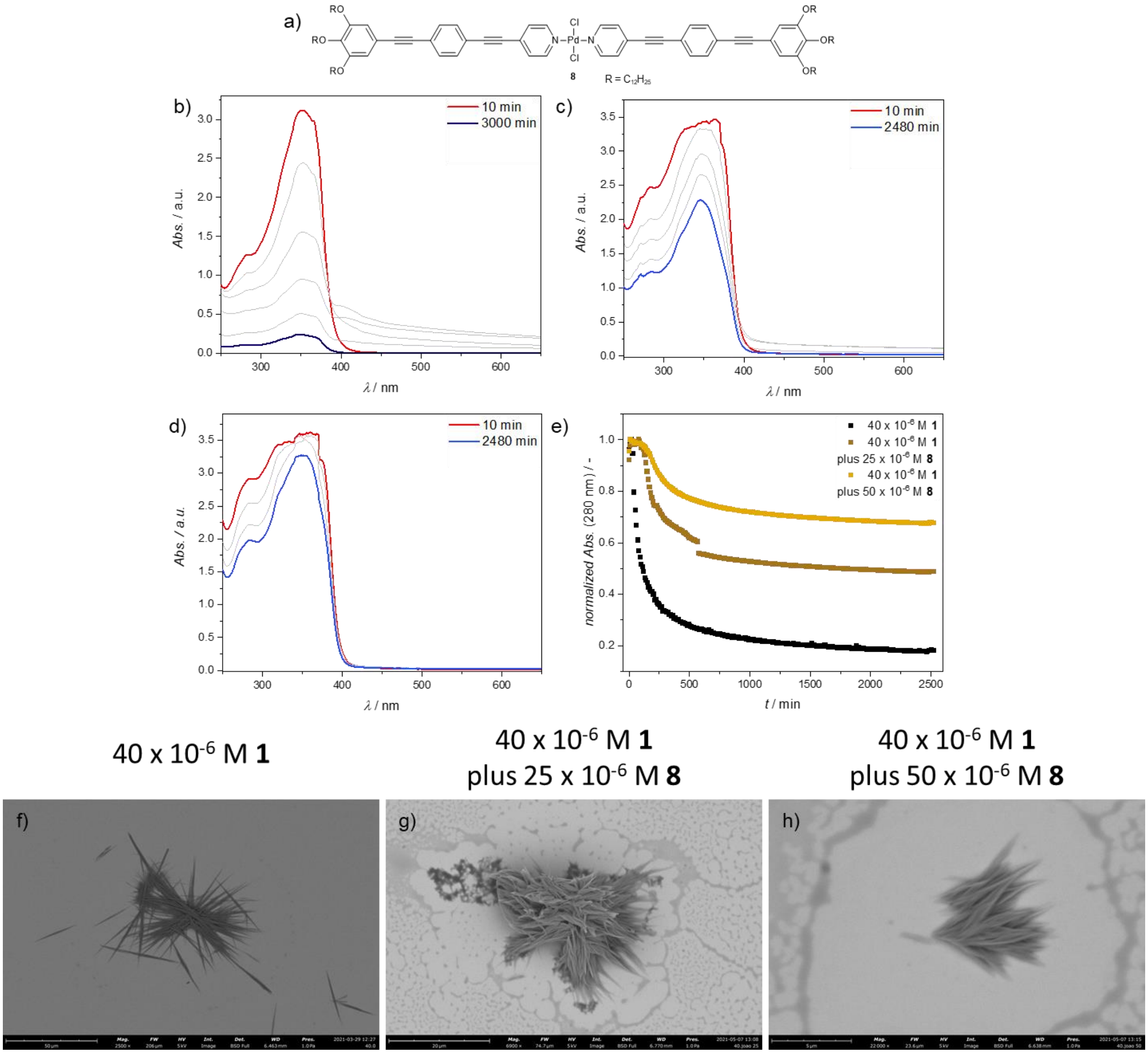

Supplementary Figure 52: a) Chemical structure of **8**. b-e) Time-dependent UV/Vis spectra of mixtures of **1** and **8** using a concentration of 40 x 10^{-6} M of **1** and varying amounts of **8** (0 (b), 25 (c) and 50 x 10^{-6} M (d)) with the time-dependent absorbance changes at λ = 280 nm depicted in (e). f-h) SEM micrographs of Agg**1** obtained after the time-dependent UV/Vis studies.

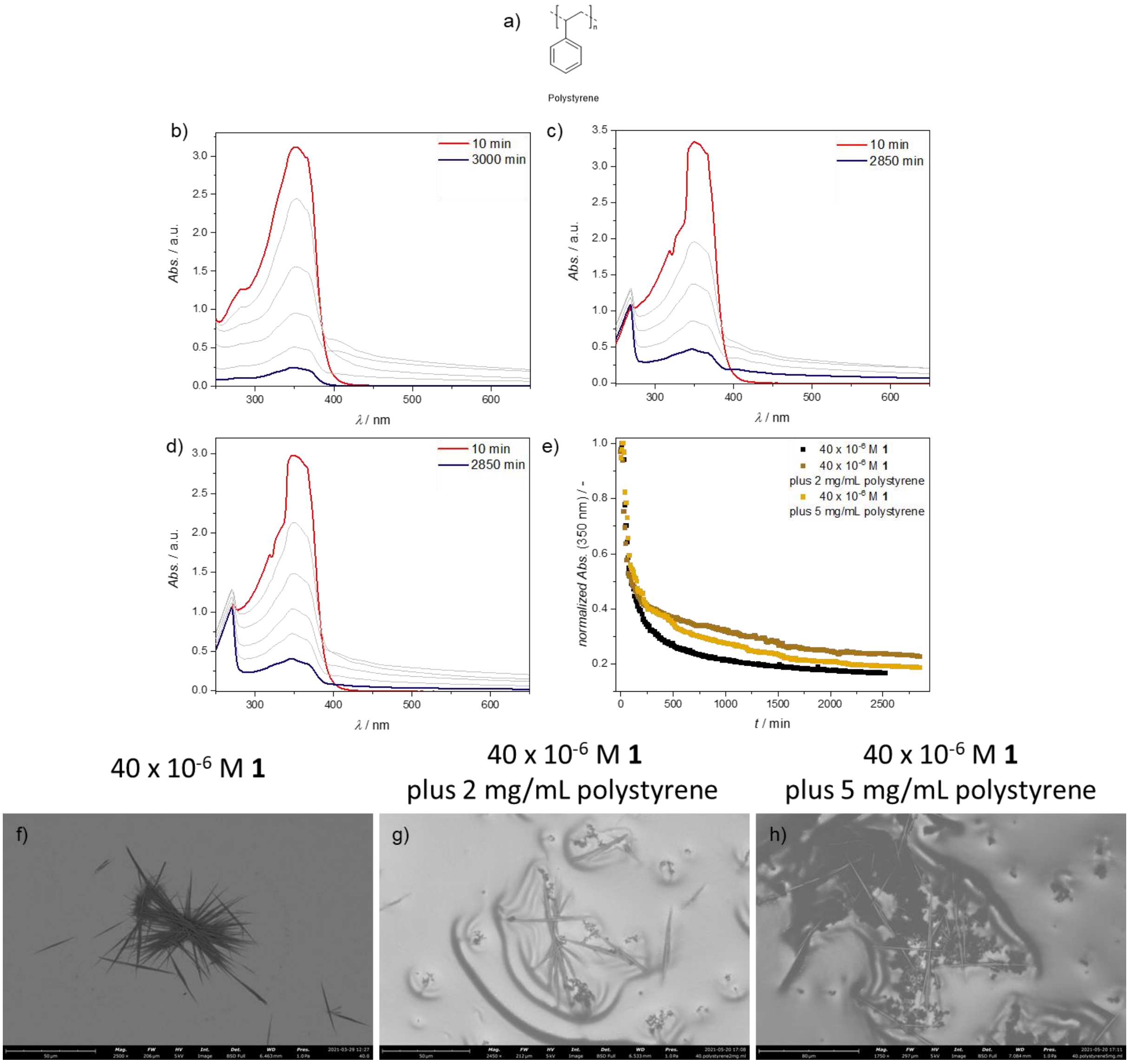

Supplementary Figure 53: a) Chemical structure of polystyrene. b-e) Time-dependent UV/Vis spectra of mixtures of **1** and polystyrene using a concentration of 40 x 10^{-6} M of **1** and varying amounts of polystyrene (0 (b), 2 (c) and 5 mg/mL (d)) with the time-dependent absorbance changes at the absorption maximum of **1** (λ = 350 nm) depicted in (e). f-h) SEM micrographs of Agg**1** obtained after the time-dependent UV/Vis studies. $MW_{polystyrene}$ = 13000; MW/Mn = 1.06.

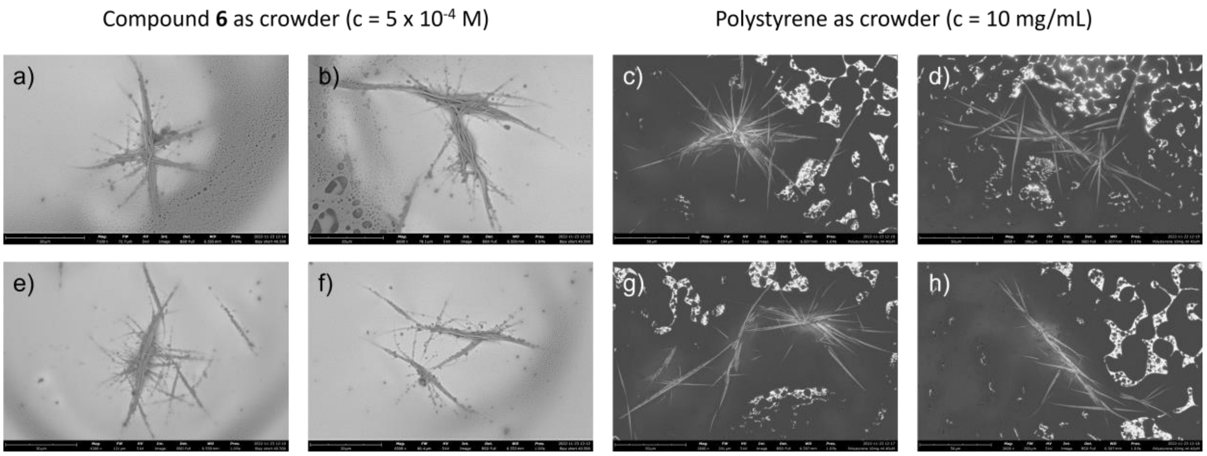

Supplementary Figure 54: SEM micrographs of Agg**1** obtained in the presence of highly elevated concentrations of **6** as a non-effective supramolecular (a,b,e,f) and polystyrene as a non-effective macromolecular (c,d,g,h) crowder using a concentration of 40 x 10^{-6} M of **1**.
